# Jumbo Bacteriophages Are Represented Within an Increasing Diversity of Environmental Viruses Infecting the Emerging Phytopathogen, *Dickeya solani*

**DOI:** 10.3389/fmicb.2018.02169

**Published:** 2018-09-12

**Authors:** Andrew Day, Jiyoon Ahn, George P. C. Salmond

**Affiliations:** Department of Biochemistry, University of Cambridge, Cambridge, United Kingdom

**Keywords:** *Dickeya solani*, bacteriophage, environmental viruses, phytopathogen, horizontal gene transfer, phage therapy, *Ackermannviridae*, jumbo bacteriophage

## Abstract

*Dickeya* species are economically important phytopathogens widespread in mainland Europe that can reduce crop yields by 25%. There are no effective environmentally-acceptable chemical systems available for diseases caused by *Dickeya*. Bacteriophages have been suggested for use in biocontrol of these pathogens in the field, and limited field trials have been conducted. To date the majority of bacteriophages capable of infecting *Dickeya solani*, one of the more aggressive species, are from the same family, the *Ackermannviridae*, many representatives of which have been shown to be unsuitable for use in the field due to their capacity for generalized transduction. Members of this family are also only capable of forming individual plaques on *D. solani*. Here we describe novel bacteriophages from environmental sources isolated on *D. solani*, including members of two other viral families; *Myoviridae* and *Podoviridae*, most of which are capable of forming plaques on multiple *Dickeya* species. Full genomic sequencing revealed that the *Myoviridae* family members form two novel clusters of jumbo bacteriophages with genomes over 250 kbp, with one cluster containing phages of another phytopathogen *Erwinia amylovora*. Transduction experiments showed that the majority of the new environmental bacteriophages are also capable of facilitating efficient horizontal gene transfer, however the single *Podoviridae* family member is not. This particular phage therefore has potential for use as a biocontrol agent against multiple species of *Dickeya*.

## 1. Introduction

The genus *Dickeya*, recently reclassified into the novel family Pectobacteriaceae (Adeolu et al., 2016), currently consists of 11 phytopathogenic species that can cause severe disease in economically important crops including tomato, orchid, and potato (Alic et al., 2017a). Until 2004, almost all European potato isolates of *Dickeya* were assigned as *Dickeya dianthicola* (Toth et al., 2011). In 2008/2009, a new clade of *Dickeya* in European potato isolates was identified (Laurila et al., 2008; Parkinson et al., 2009; Sławiak et al., 2009) and in 2014 a new species was proposed; *Dickeya solani* (van der Wolf et al., 2014).

*Dickeya solani* is able to spread more easily through the plant vascular system and survive at higher temperatures than *D. dianthicola* (Toth et al., 2011). It is currently the predominant potato pathogen in Europe, with reductions in yield of up to 25% reported in potatoes exposed to *Dickeya* species (Tsror et al., 2009). Whilst there have been isolated cases of *D. solani* reported in England and Wales since 2007 (Cahill et al., 2010), these were all found in crops originating from outside of the UK (Toth et al., 2016). It is currently yet to become established in the UK, and to mitigate the significant economic cost inflicted by this virulent phytopathogen, the Scottish government has introduced specific legislation aimed at preventing the establishment of *D. solani* in its seed industry (Mansfield et al., 2012).

The significant economic costs inflicted by *Dickeya* species have stimulated research interest in methods for control of these virulent phytopathogens. Bacterial viruses (bacteriophages; phages) have been suggested as potential tools for biocontrol due to their specificity, environmental persistence and biological “organic” nature (Iriarte et al., 2007; Czajkowski et al., 2017; Svircev et al., 2018). Several studies have isolated phages capable of infecting *Dickeya* species (Adriaenssens et al., 2012c; Czajkowski et al., 2014a,b, 2015a; Matilla et al., 2014; Alič et al., 2017b; Day et al., 2017). Their potential use as biocontrol agents has been trialed both in the lab and in the field (Adriaenssens et al., 2012c) and these studies showed a partially “therapeutic” outcome with reduced crop losses. There is a commercial product available, Biolyse™, from APS Biocontrol Ltd that is a phage cocktail able to target *Pectobacterium* as well as *Dickeya* species. Designed as a washing solution for potatoes during factory processing, to our knowledge it is the first, and currently the only, commercial *Dickeya*-targeting biocontrol product. It has been reported that Biolyse™ has been used by the UK supermarket chain Tesco (Branston, 2012). The identities of the phages contained within this cocktail however have not been reported.

All of the *Dickeya* phages isolated so far, and 96% of all known phages, are members of the order *Caudovirales* (Fokine and Rossmann, 2014), which currently consists of four families. Apart from the *Siphoviridae* family member BF-CIM1/14 recently described by Alič et al. (2017b) and three *Podoviridae* family members reported in our recent publication (Day et al., 2017), the vast majority of *D. solani* phages characterized so far share a high degree of similarity and have been designated members of the *Ackermannviridae* family (formerly known as the *Vi1virus* genus; Adriaenssens et al., 2018) based on morphology and genomic comparisons. A summary of these phages is shown in Table 1. This has generated research interest, as these phages have been isolated from both soil and water samples and in three separate European countries; Belgium, Poland, and the United Kingdom. Host range testing has shown that the phages isolated in Belgium and the majority isolated in the UK are capable of forming plaques on strains of *D. solani* only (Adriaenssens et al., 2012c; Day et al., 2017). The phages isolated in Poland are reported to infect multiple species of *Dickeya* and *Pectobacterium* (Czajkowski et al., 2014a,b, 2015a), however, host range testing to the level of individual plaque formation has not been reported—an important criterion that allows exclusion of false positives (Khan Mirzaei and Nilsson, 2015). The high degree of morphological and genomic similarity between these phages and the other *Ackermannviridae* family members makes the reported broader host range that spans genera an intriguing prospect, assuming plaque formation data supporting this broader host range can be confirmed.

**Table 1:** Members of the *Ackermannviridae* family isolated on *Dickeya solani*.

**Bacteriophage**	**Isolation**	**Location**	**Genome size (bp)**	**References**
LIMEstone1	2008	Belgium (soil)	152,247	Adriaenssens et al., 2012c
D5	2012	Poland (soil)	155,346	Czajkowski et al., 2014b
PD10.3	2013	Poland (soil)	156,113^*^	Czajkowski et al., 2015a
PD23.1	2013	Poland (soil)	153,365^*^	Czajkowski et al., 2015a
D3	2013	Poland (soil)	152,308	Czajkowski et al., 2015b
XF4	2013	UK (waterway)	151,519	Day et al., 2017
JA15	2014	UK (waterway)	153,757	Day et al., 2017

* *Genomes are marked incomplete, largest scaffold is reported and exhibits 99% DNA identity to LIMEstone1*.

Sixty-seven phages were described in our recent publication (Day et al., 2017), 59 of which were only capable of forming plaques on *D. solani* species. When two were genomically sequenced they showed a high degree of similarity with the previously published *D. solani* phages of the *Ackermannviridae* family. The remaining eight phages were capable of forming plaques on other species of *Dickeya*, including *Dickeya zeae, Dickeya chrysanthemi*, and *Dickeya paradisiaca*. These particular phages warranted further investigation, as an expanded host range can be helpful for further application in phage therapy. The aim of this study therefore was to genomically sequence these phages to determine their similarity to previously published *D. solani* phages and their suitability for use in phage therapy.

## 2. Results

### 2.1. Host range and plaque morphology

The plaques of the previously described *D. solani* phages that are members of the *Ackermannviridae* family have tended to be clear, defined, and easy to distinguish from the bacterial top lawn (Adriaenssens et al., 2012c; Czajkowski et al., 2015a). This is the case for the other *Ackermannviridae* family members isolated in this laboratory, and is also true of one of the broader host range phages JA10. However, the other seven have an indistinct, turbid plaque morphology that is often hard to distinguish from the bacterial top lawn (data not shown). We believe this to be the reason why the host range of the eight *D. solani* phages shown in Table 2 is different from the host range reported in the previous publication (Day et al., 2017). This first became apparent due to confusing results generated by related, unpublished experiments that suggested a variation in the host range from that published in our previous paper (Day et al., 2017). Rigorous retesting has confirmed that the host range data presented in Table 2 are accurate and that the previous interpretations were incorrect. The efficiency of plating data in Table 2 shows that most of the phages are able to adsorb at a similar efficiency to all species of *Dickeya*, apart from JA29 which has an efficiency 10^−4^ lower on *D. paradisiaca* and *D. dadantii* subsp. *dieffenbachiae*.

**Table 2:** Broader host range of eight phages capable of infecting other species of *Dickeya* as well as *Dickeya solani*.

***Dickeya* species**	**JA10**	**JA11, 31, 32**,	**JA13**	**JA29**
		**33 and 37**		
*D. solani*	1	1	1	1
*D. dadantii* subsp. *dieffenbachiae*	1.00 × 10^−1^	6.50 × 10^−1^ ± 6.30 × 10^−1^	7.50 × 10^0^	5.40 × 10^−4^
*D. paradisiaca*	–	6.30 × 10^−1^ ± 1.39 × 10^−1^	5.80 × 10^−1^	3.5 × 10^−5^
*D. dianthicola*	–	5.50 × 10^−1^ ± 3.25 × 10^−1^	–	–
*D. zeae*	–	1.12 × 10^0^ ± 5.80 × 10^−1^	4.40 × 10^−1^	–
*D. chrysanthemi*	1.30 × 10^−1^	–	–	–

*Efficiency of plating is relative and is calculated by dividing the titre of the phage on the host question by the titer of the phage on the original host D. solani. –Denotes no observable plaque formation of the phage on this host*.

### 2.2. Morphological classification

**Figure 1:**
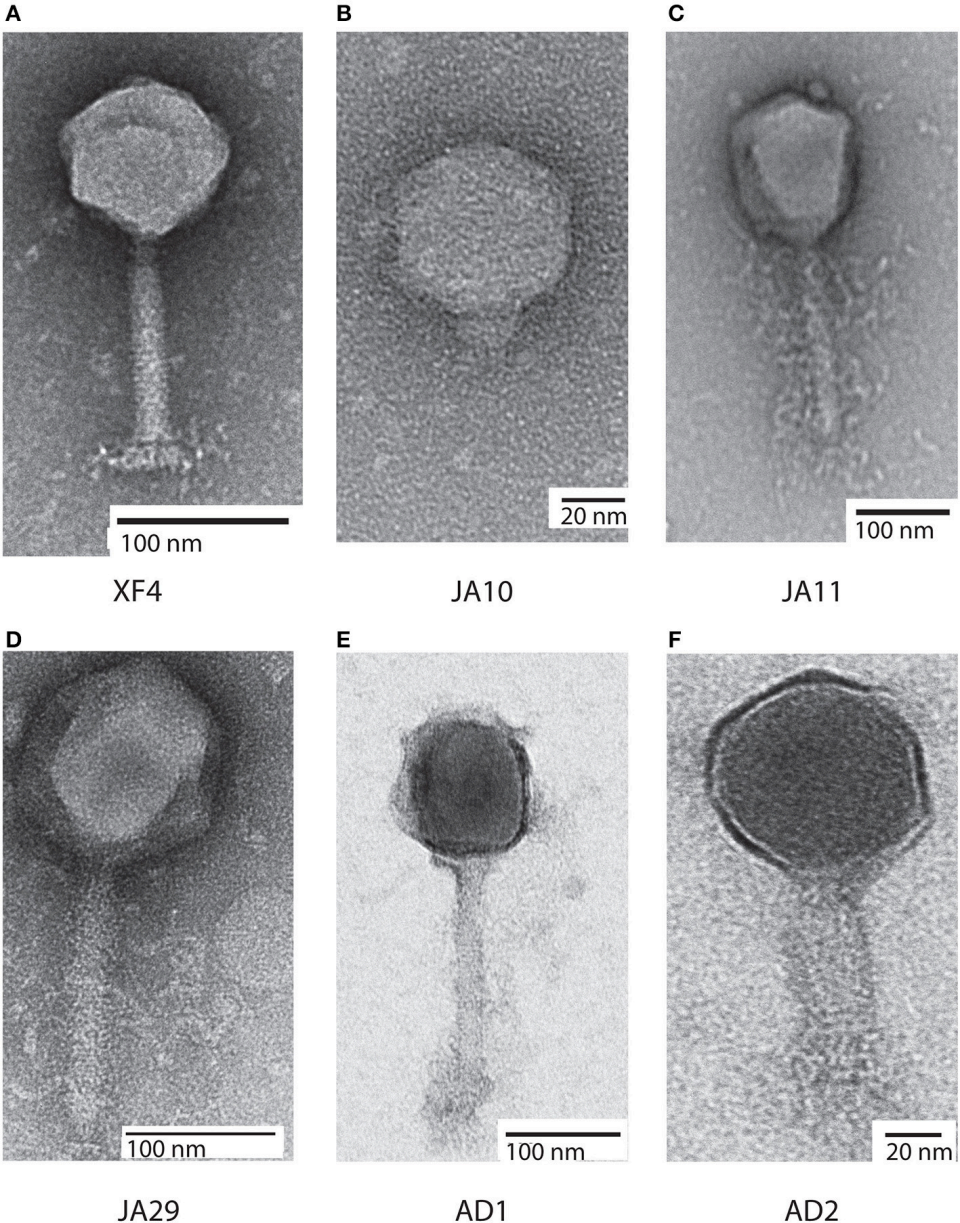
Transmission electron micrographs of six *Dickeya solani* phages. XF4 **(A)** is a member of the *Ackermannviridae* family, exhibiting an icosahedral head of around 90 nm, a tail length of 110 nm and tail spikes at the base of the tail. JA10 **(B)** is a member of the *Podoviridae* family, characterized by a short stubby tail. JA11, JA29, and AD1 **(C–E)** are members of the *Myoviridae* family with unclear tail appendages. AD2 **(F)** has a similar morphology to XF4 and has a partially-contracted tail.

Bacteriophages have traditionally been classified based on morphological characteristics viewed under electron microscopy (Ackermann, 2012). The majority of *D. solani* phages isolated to date are members of the *Ackermannviridae* family, which share common morphological characteristics. A representative of this family, XF4, is shown in Figure 1A. These phages possess an icosahedral head with a diameter of around 90 nm, a contractile tail around 110 nm in length and structures at the base of the tail that have been described as “stars” or “prongs” and have been identified as tail spikes (Adriaenssens et al., 2012a). Apart from these tail spikes, this is classical morphology of the phage family *Myoviridae*, therefore the combination of a contractile tail and tail spikes are the morphological markers of the family *Ackermannviridae*.

All eight of the expanded host range phages were viewed under transmission electron microscopy. Seven of them had an icosahedral head and long tail, with the structures at the base of the tail remaining unclear, with two representatives shown in Figures 1C,D. This marks them as either members of the *Myoviridae* or *Ackermannviridae* families, however, these phages were significantly larger than the previously viewed members of the *Ackermannviridae* family, which can be seen by comparing Figure 1A with Figure 1D. The head diameters were over 120 nm, with a tail length of around 150 nm, which suggested that these phages were not *Ackermannviridae* family members.

The indistinct morphology of the tail appendages of the JA jumbo phages (best seen in Figure 1C) has also been identified in other phages. When first described in the *Escherichia* phage 121Q (Ackermann and Nguyen, 1983) this morphology was presumed to be an artifact of microscopy involving damage to the tail. It was also thought that the dimensions, at the time reported to be a head diameter of 150 nm and a tail length of 165 nm, were overstated. However, this morphology has since been directly reported in the *Pseudomonas putida* phage Lu11 (Adriaenssens et al., 2012b), the *Pectobacterium carotovorum* phage CBB (Buttimer et al., 2017), and the *Erwinia amylovora* phage Y3 (Buttimer et al., in press), and has been dubbed the “hairy *Myoviridae*” morphology (Buttimer et al., 2017).

Unexpectedly, as can be seen in Figure 1B, the phage JA10 could be classified as a member of the *Podoviridae* family when imaged, characterized by an icosahedral head and a short non-contractile tail. Whilst this is not the first member of this family that we have isolated (Day et al., 2017), this is the first isolate we have studied in further depth. The genome of JA10 was therefore sequenced to investigate the similarity between it and previously published *Dickeya*-infecting *Podoviridae* family members (Alič et al., 2017b).

### 2.3. Genome sequence of *Podoviridae* family member JA10

**Figure 2:**
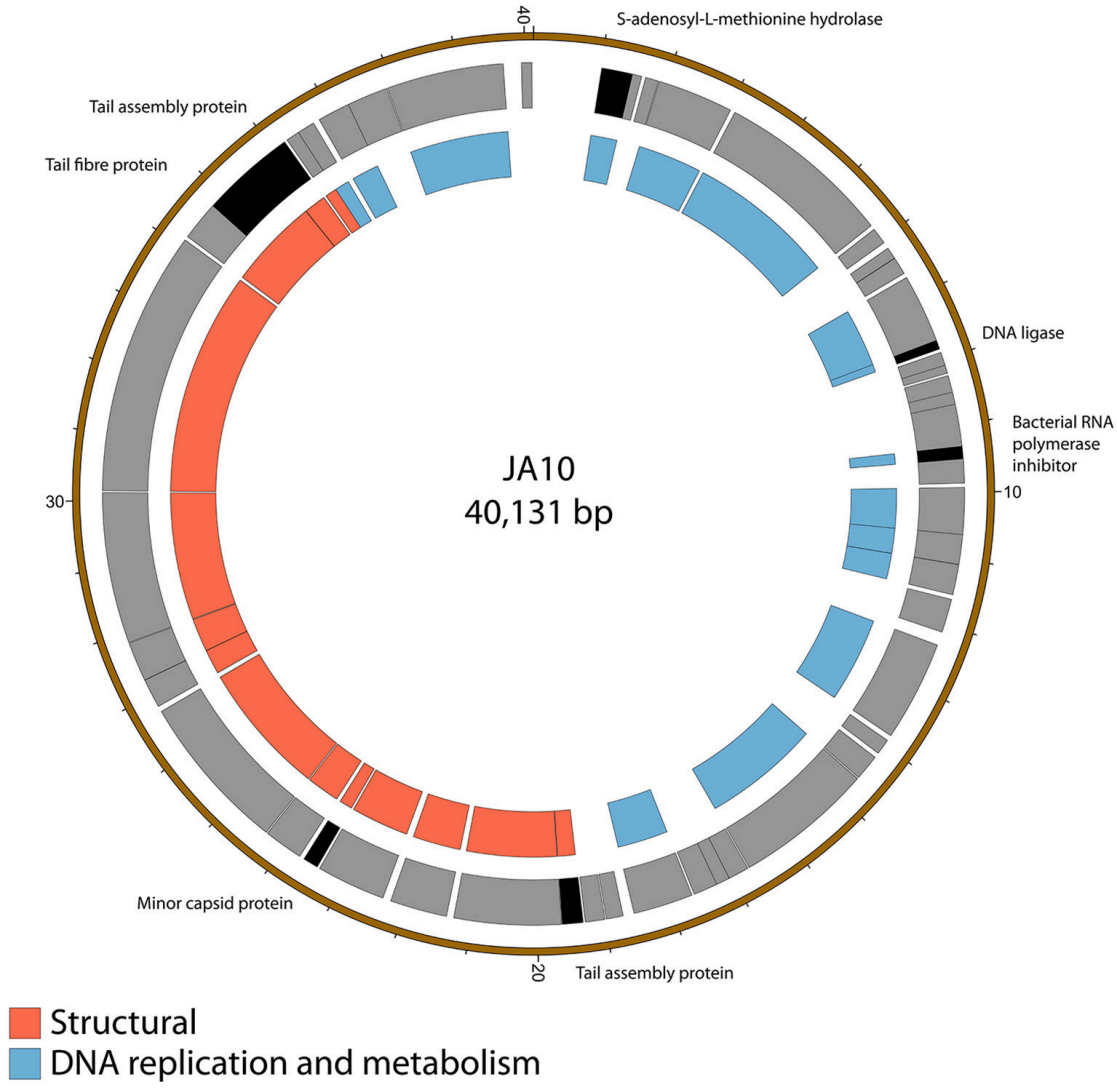
Map of the genome of JA10. The outer gray ring marks open reading frames, with those highlighted in black discussed in more detail in the text, whilst the inner ring categorizes the proposed functions of these genes. The genome map was generated using Circos.

The genome of JA10 is 40,131 bp, has 50 predicted genes, and is shown in Figure 2. The closest match in the database is an as yet unpublished *D. solani* phage Ninurta (Genbank reference: MH059639) isolated from organic waste in Denmark that shares 95% DNA identity with JA10. The closest published phage is the *Pectobacterium parmentieri* phage PP74 isolated from potato washing waste water in Russia in 2015 (Kabanova et al., 2018), which shares less than 14% nucleotide identity. It shares no DNA sequence identity with the other sequenced *Dickeya*-infecting *Podoviridae* family member BF25/12 (Alič et al., 2017b). PP74 has been designated as a T7-like virus and a member of the *Autographivirinae* subfamily, with a conserved core genome. A translated nucleotide comparison of JA10 with the type phage T7 showed that most of the predicted genes are conserved (data not shown), including almost all the genes with a proposed function, such as the T3/T7-like RNA polymerase, structural capsid genes and DNA packaging machinery. JA10 is therefore also a member of the *Autographivirinae* subfamily.

There are several putative proteins encoded in the JA10 genome that do not have significant homology in the T7 genome and these are highlighted in Figure 2. Four of these seven putative proteins, the S-adenosyl-L-methionine hydrolase, bacterial RNA polymerase inhibitor, minor capsid protein, and the first tail assembly protein, have a protein of similar function encoded at this position in T7. The variation between JA10 and T7 in these putative proteins is therefore likely a determinant of host specificity. The marked tail fiber protein, which share a common N-terminal region but differ at the C-terminus between the two phages, is also likely a contributor to host range specificity. The DNA ligase highlighted in Figure 2 neighbors a conserved ligase and consists of fewer than 200 amino acids. This is therefore a possible result of a recombination duplication event and may not be a functional protein. The final tail assembly protein, close to the end of the JA10 genome, has no functional homologue in T7.

### 2.4. Novel jumbo “hairy *Myoviridae*” phages

Sequencing of the other seven genomes showed that, although the phages were isolated independently, several shared 100% identity at the nucleotide level. JA11, 31, and 32 grouped together, as did JA33 and 37. JA31, 32, and 37 were therefore excluded from further analysis. The genome size for the four remaining phages is between 253 and 256 kbp. A summary of the characteristics of these genomes, as well as JA10, is shown in Table 3. These phages are therefore jumbo phages, defined as phages with a genome over 200 kbp (Yuan and Gao, 2017). The genomes are significantly larger than the conserved size of the *Ackermannviridae* family genomes, which are around 150 kbp, and larger than most sequenced phages. As of July 2018, there were 9,351 recorded phage genome sequences in Genbank (http://millardlab.org/bioinformatics/bacteriophage-genomes/) and these JA phages would be the 62nd–65th largest sequenced. Many of the over 300 predicted open reading frames in each genome do not match any known genes; the majority of those that do share identity with known genes are from the *E. amylovora* phages Yoloswag (Esplin et al., 2017) and Y3 (Buttimer et al., in press). These are largely structural genes and genes involved in DNA metabolism and replication.

**Table 3:** Summary of the genomes of the broader host range phages.

**Phage**	**Genome size (bp)**	**GC content (%)**	**Open reading frames**
JA10	40,131	51.5	50
JA11	255,356	44.5	321
JA13	254,061	44.5	323
JA29	253,323	43.8	318
JA33	255,356	44.5	321

### 2.5. Variation within the JA phages

**Figure 3:**
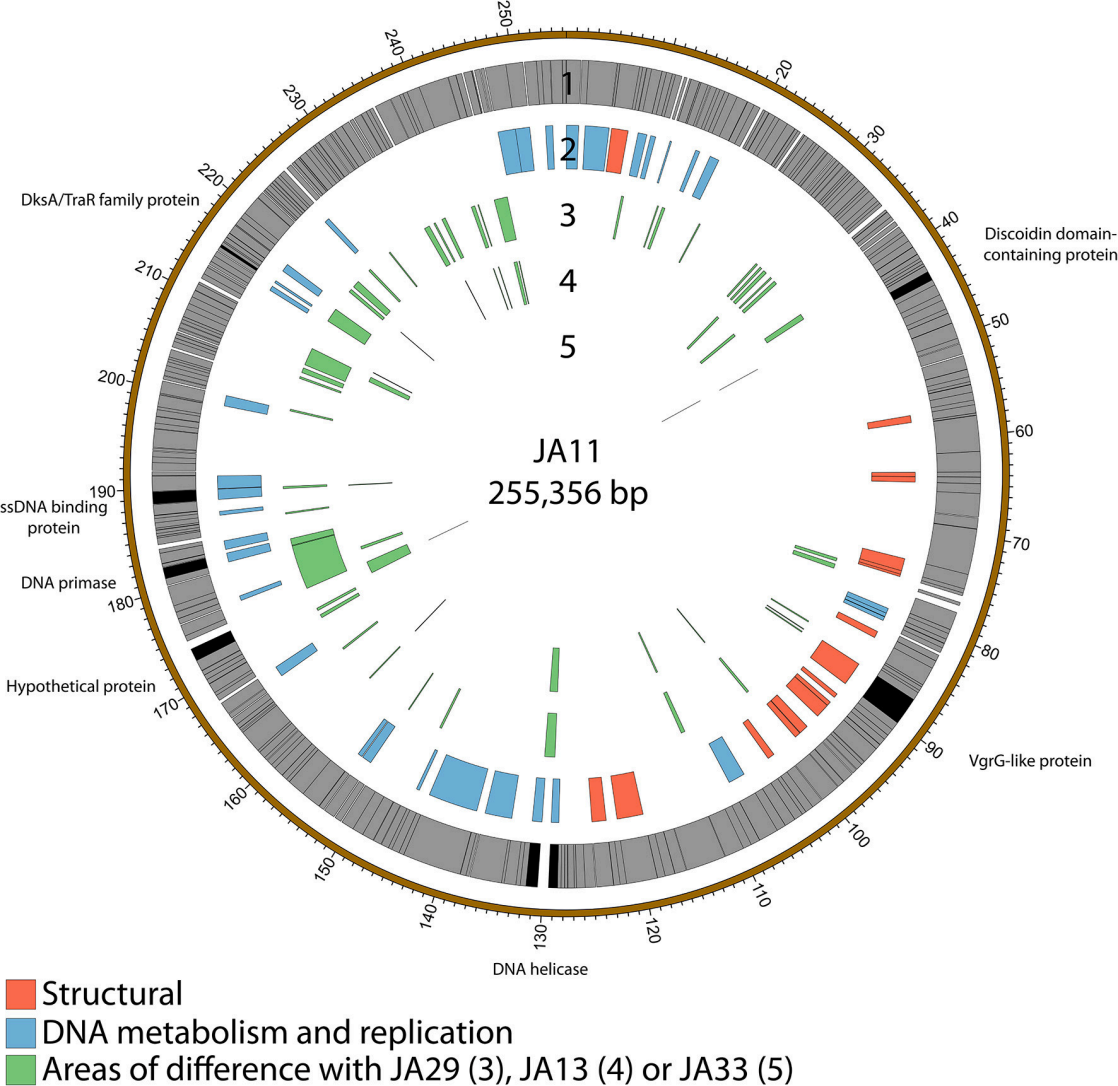
Map of the genome of JA11. The outer gray ring marks open reading frames, with those highlighted in black discussed in more detail in the text. The second ring categorizes the proposed functions of these genes, whilst the inner rings highlight the areas of the genome that differ from the genomes of JA29, JA13, and JA33 (third to fifth ring, respectively). The genome map was generated using Circos.

The gene order of the four JA jumbo phages is largely conserved. Over three quarters of the predicted ORFs are annotated as encoding hypothetical proteins, and many of the differences between the phages are contained within these ORFs as shown in Figure 3. JA29 is the most dissimilar to the others, sharing 86% nucleotide identity with JA11, whereas the nucleotide identity between JA11 and JA13 is 95%. JA11 and JA33 are 99% identical, with the major difference being the insertion of 126 bp in both genomes at different positions, and of different sequences. These insertions are in non-coding regions however, therefore their biological relevance is unclear. The only other differences are in two putative proteins. One is a hypothetical protein, whilst the other contains a putative discoidin domain, with the substitution between JA11 and JA33 (alanine to threonine) in the middle of the domain. Discoidin domains are present in eukaryotic agglutination factors and therefore the possible biological role for this in a phage genome, and the effect of the substitution, is not immediately obvious.

JA11, 13, and 29 have differing host ranges, as listed in Table 2. To investigate whether this was caused by variations within the tail fibers of the phages, the amino acid sequences of each of the three putative tail fiber proteins was compared between the phages. JA11 and JA13 possess identical tail fibers, whereas JA29 shows variations of several amino acids in each protein, as listed in Table 4. Whilst these variations could explain the differing host range of JA11 and JA29, it does not explain the difference in host range observed for JA11 and JA13.

**Table 4:** Summary of the annotated genes which differ between JA11, JA13, and JA29.

**Function**	**Gene**	**Length**	**Differences with JA11**
Tail fiber one	JA11_90	272	
	JA13_090		0
	JA29_093		6
Tail fiber two	JA11_94	164	
	JA13_095		0
	JA29_096		7
Tail fiber three	JA11_95	210	
	JA13_096		0
	JA29_098		1
DNA primase	JA11_208	350	
	JA13_208		0
	JA29_210		7
DNA helicase	JA11_155 + 156	^*^	
	JA13_156		^*^
	JA29_158		^*^
DksA/TraR family protein	JA11_264	85	
	JA13_267		1
	JA29_265		5
ssDNA binding protein	JA11_221	402	
	JA13_222		1
	JA29_223		32
VgrG-like protein	JA11_117	931	
	JA13_118		1
	JA29_120		87

* *See Figure 4*.

Whilst most of the differences between the phages are located in genes with no predicted function, there are a few annotated that are present in all of the JA phages. These include encoding a DNA helicase, two potential transcription factors and one structural protein, all highlighted in Figure 3 and listed in Table 4.

**Figure 4:**
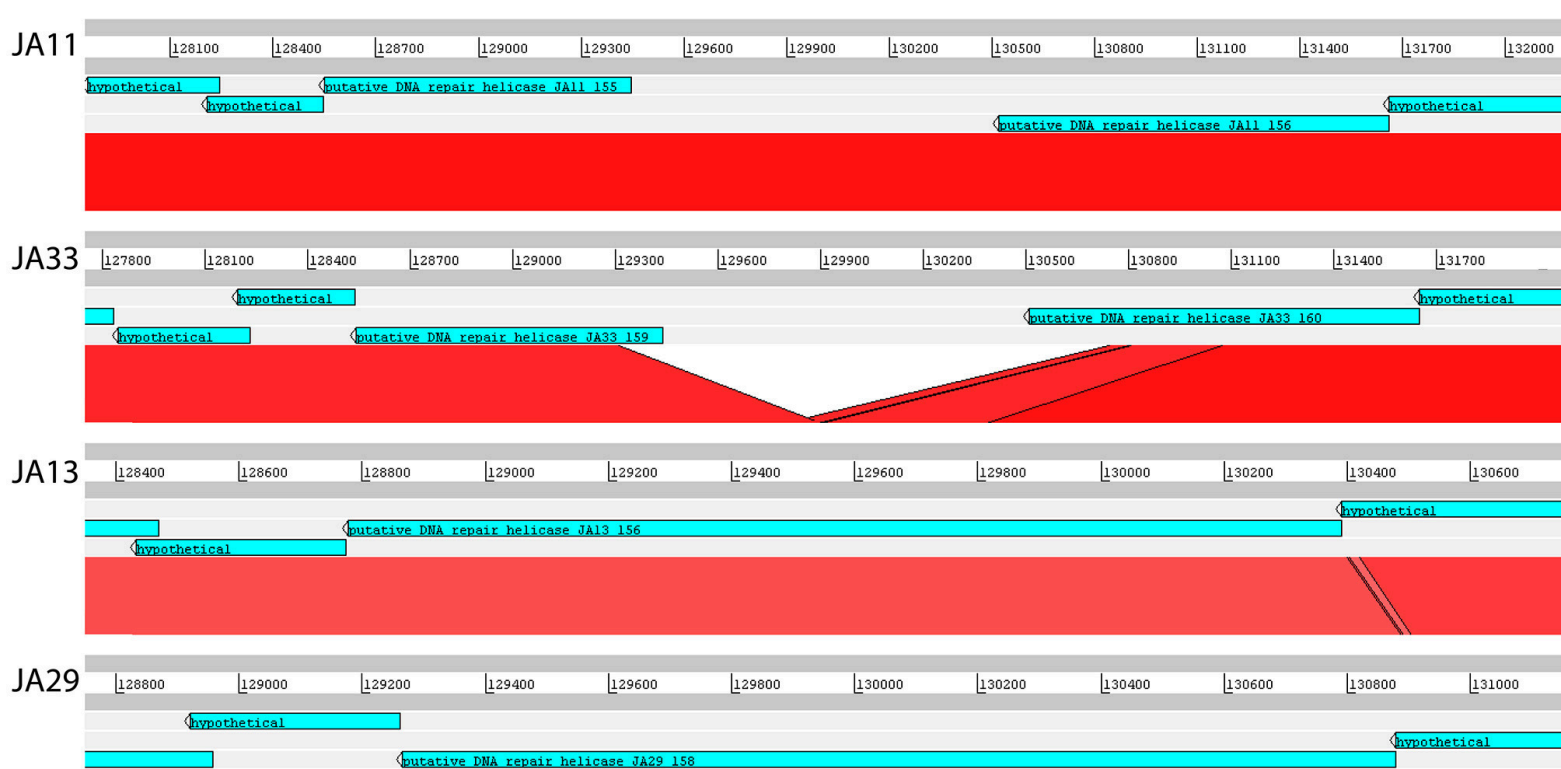
Translated nucleotide comparison of a putative DNA helicase between JA11 (top), JA33 (second), JA13 (third), and JA29 (bottom). Red bars mark areas of amino acid identity, with darker colors showing higher identity. Figure produced using the Artemis Comparison Tool.

There are variations in two DNA related genes: a DNA primase and a DNA helicase. The helicase shows the most variation between the phages, as it appears to have undergone insertion or deletion between some of the phages. A comparison of this region of the genome can be seen in Figure 4. There are two ORFs annotated as putative helicases in JA11 and JA33, which both share homology with one ORF in JA13 and JA29. Whether the two ORFs are able to function independently as a helicase, or whether this duplication has rendered them non-functional, is unknown. A DksA/TraR family protein and a ssDNA binding protein, both likely transcription factors, both differ in one amino acid between JA11 and JA13, and share lower identity with the JA29 homolog, particularly the ssDNA binding protein, which differs in 32 positions. A VgrG-like family protein, a component of the T6SS thought to be phage-derived as it is capable of assembling into a structure similar to a phage tail spike (Cianfanelli et al., 2016) shares, at best, only 33% amino acid identity with the closest hit in the *E. amylovora* phage Y3, and so these may define a relatively novel VgrG-like protein group. JA11 and JA13 differ by one conservative substitution in this protein, whilst JA29 differs in 87 amino acids, 13 of which are conservative substitutions. It is possible that differences in this predicted protein are a contributing factor to the differing host range of these phages.

The biological significance of the differences observed between the four JA phages is currently unclear. It is somewhat surprising to find variations in genes involved in transcription initiation and DNA replication, as the genomes of these phages are relatively similar in both size and GC content, as summarized in Table 3. It is therefore possible that these differences do not significantly alter the function of these proteins. The variation in the VgrG-like proteins is more logical, as the different host ranges of these phages may be related to differences in tail spikes and other host recognition factors. To determine the impact of these differences, and to investigate why JA11 and JA13 have a different host range despite having apparently identical tail fibers, requires further experimental work.

### 2.6. More recent isolates: AD phages

All of the JA phages were isolated from the River Cam in November 2014. Isolation of XF phages from the same location a year earlier produced mainly members of the *Ackermannviridae* family and a few *Podoviridae* family members (Day et al., 2017). Whilst there is clearly some maintenance of viral populations, as members of the two families have been isolated on both occasions, the jumbo phages presented here are a novel grouping. To gain further insight into the viral populations in the River Cam, further samples were taken in October 2017. Two phages were isolated on *D. solani* and are named AD1 and AD2. Whilst both were only capable of forming plaques on *D. solani* and not on strains of other species, microscopy showed that they had differing morphologies. AD1 (Figure 1E) appears to have a “hairy *Myoviridae*” morphology similar to that of the JA jumbo phages, with a head diameter of 120 nm, tail length of 150 nm, and unclear structures at the base of the tail. AD2 on the other hand (Figure 1F) has a head diameter of 90 nm and a (potentially partially-contracted) 70 nm tail, leading to a tentative classification as a member of the *Ackermannviridae* family. The structures at the end of the tail are inconclusive.

**Figure 5:**
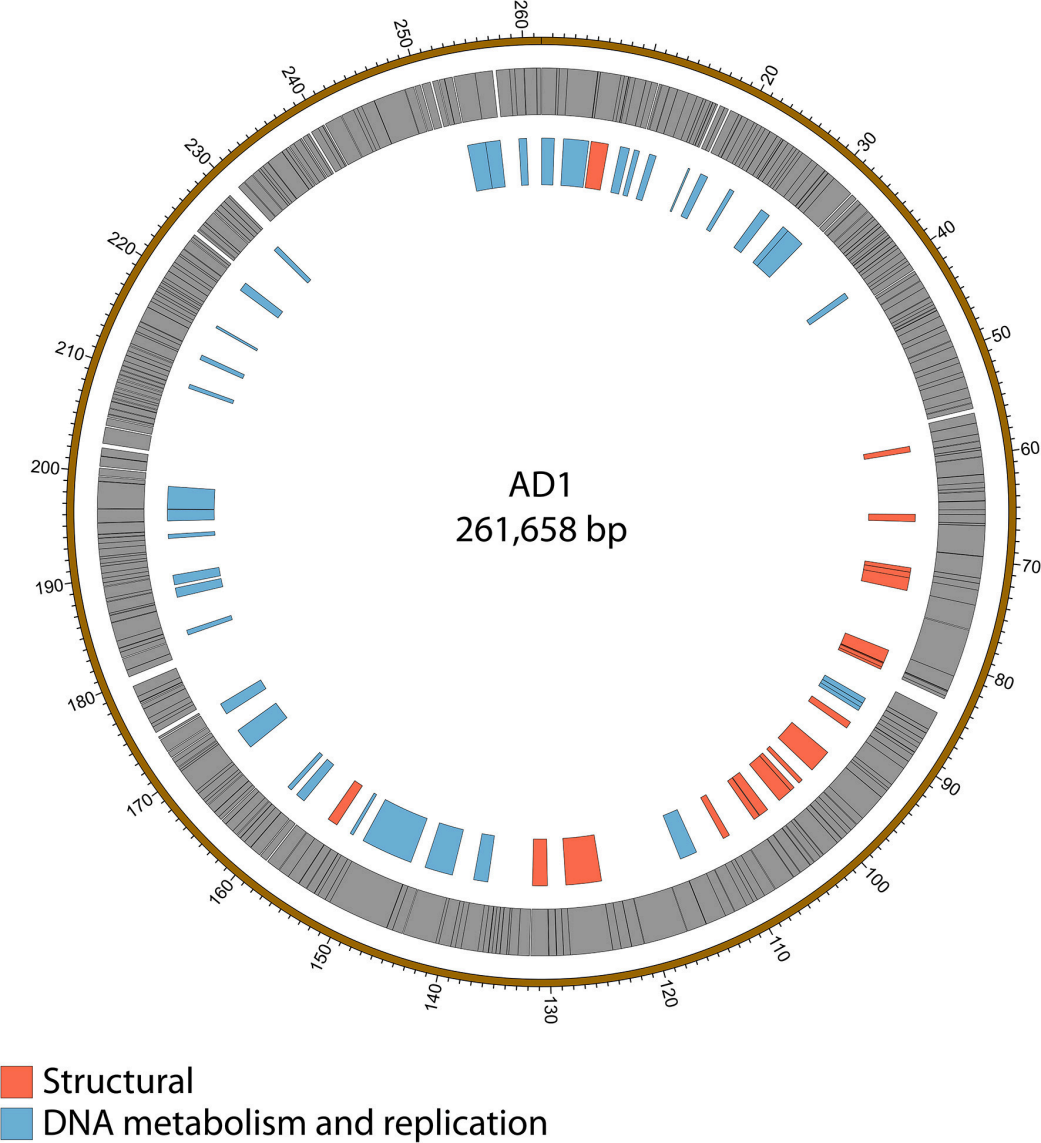
Map of the genome of AD1. The outer gray ring marks open reading frames whilst the inner ring categorizes the proposed functions of these genes. The genome map was generated using Circos.

**Figure 6:**
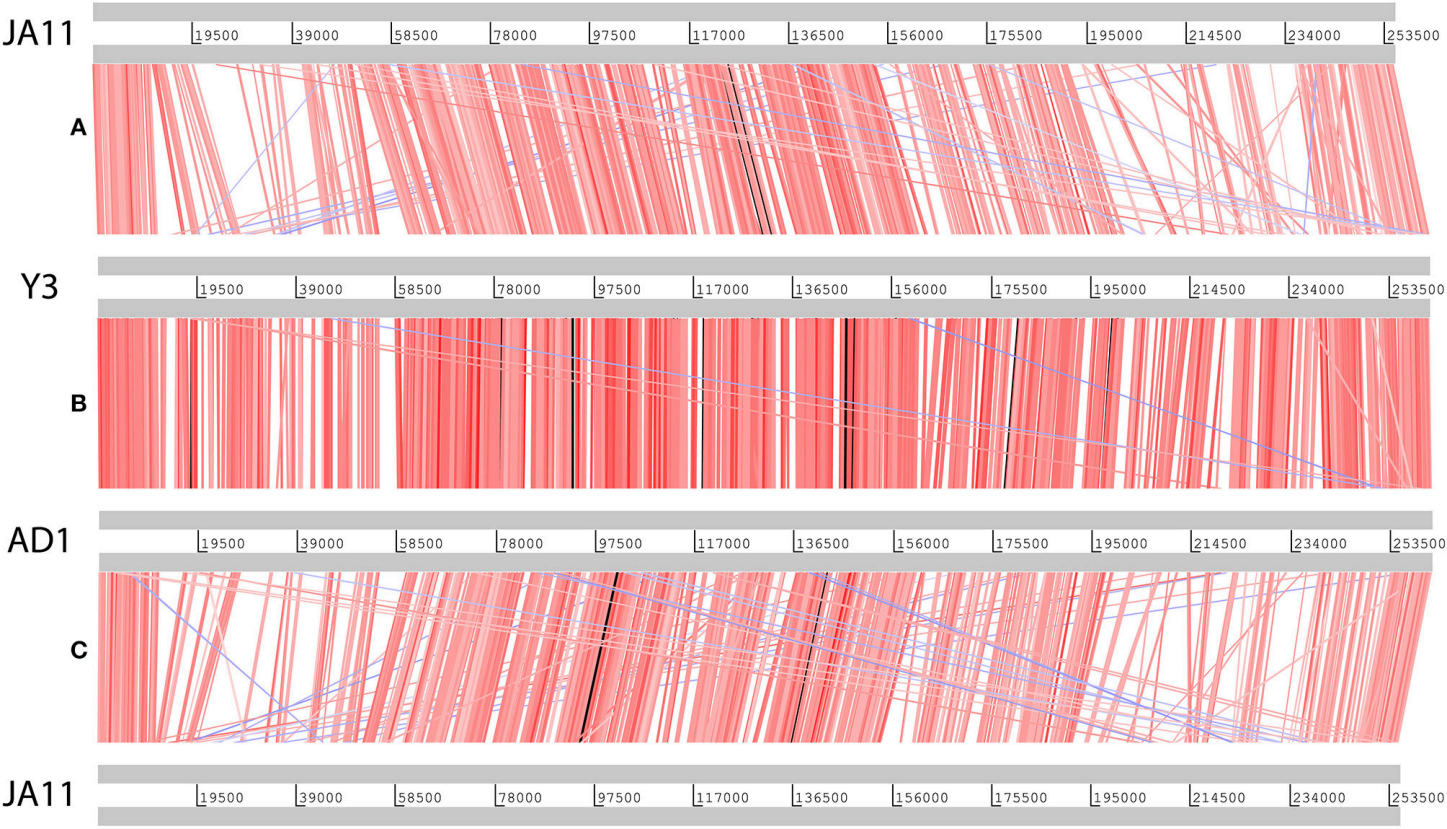
Translated nucleotide comparison of the genomes of JA11 **(A)**, Y3 **(B)**, and AD1 **(C)**. Red bars mark areas of amino acid identity, with darker colors showing higher identity. Blue bars highlight areas of inversion. Figure produced using the Artemis Comparison Tool.

Genome sequencing of the two AD phages showed that, as suggested by microscopy, AD2 is a member of the *Ackermannviridae* family. It shares 99% nucleotide identity with previously published *D. solani Ackermannviridae* such as XF4 and LIMEstone1 (Day et al., 2017), although full coverage of the genome was not achieved (data not shown). AD1, as expected, has a large genome of 261,658 bp, confirming that it is a jumbo phage, shown in Figure 5. However, unexpectedly, it has low nucleotide sequence identity with the JA jumbo phages, despite sharing a similar gene order. In fact, a translated nucleotide comparison of JA11 and AD1, as shown in Figure 6C, shows that JA11 is about as similar to AD1 as it is to Y3 (Figure 6A), and a comparison of AD1 and Y3 (Figure 6B) shows them to be more similar to each other than to JA11 at the amino acid level.

### 2.7. Phylogeny of the “hairy *Myoviridae*” phages

**Figure 7:**
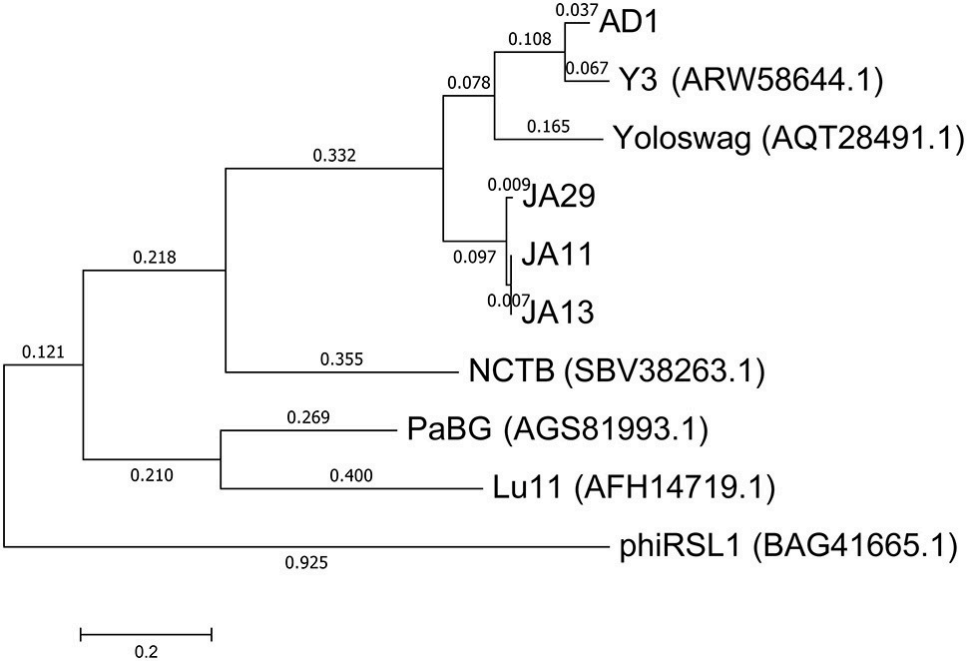
Phylogenetic tree of the large terminase subunit constructed using the Maximum Likelihood method in MEGA. All positions containing gaps and missing data were eliminated, with 642 positions in the final dataset. The tree shown has the highest log likelihood (−8677.52) and is drawn to scale, with branch lengths measured in the number of substitutions per site.

In their recent publication, Buttimer et al. discussed the phylogenetic position of Y3 considering its low level of nucleotide identity to existing genomes (Buttimer et al., in press). Two potential subfamilies within the “hairy *Myoviridae*” have emerged; the Rak2-like phages, which includes the previously mentioned *Pectobacterium* phage CBB (Buttimer et al., 2017), and the as yet unnamed subfamily that encompasses the phage discussed here. This was established as it was found that Y3 had homologs including terminase, polymerase and helicase genes in several other phages reported or suspected to have the “hairy *Myoviridae*” morphology. A comparison of the large terminase subunit of these phages with those reported here shows clear clustering, and can be seen in Figure 7. As expected, the three JA phages cluster tightly with little variation between them. As reported by Buttimer et al. the *Pseudomonas*-infecting phages PaBG (Sykilinda et al., 2014) and Lu11 (Adriaenssens et al., 2012b) form a clade, whilst the *Ralstonia solanacearum* phage phiRSL1 (Yamada et al., 2010) and the metagenomically-derived NCTB (Pfreundt et al., 2017) are single nodes within the tree. As suggested by the translated nucleotide comparison in Figure 6B, Y3 and AD1 form a clade that puts AD1 closer to *Erwinia*-infecting phages than to the other *D. solani* phages. Intriguingly, AD1 is placed closer phylogenetically to Y3 than the other *E. amylovora*-infecting phage Yoloswag (Esplin et al., 2017). The same clustering is seen when using the sequence of the tail sheath proteins of the phages. All of the phages except phiRSL1 have two annotated tail sheath proteins, and the same phylogeny is seen with both (data not shown).

**Figure 8:**
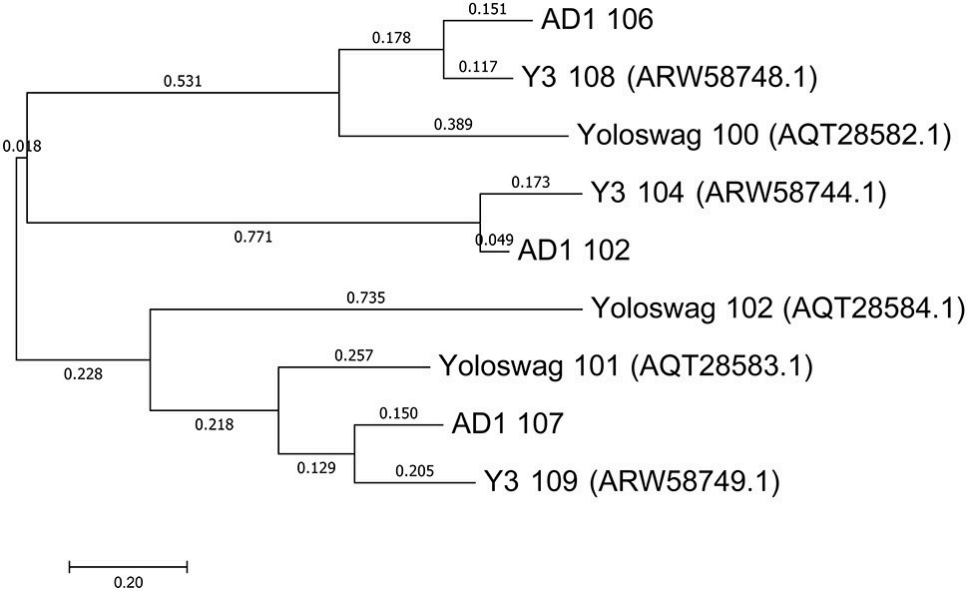
Phylogenetic tree of three tail fiber proteins constructed using the Maximum Likelihood method in MEGA. All positions containing gaps and missing data were eliminated, with 158 positions in the final dataset. The tree shown has the highest log likelihood (−2426.84) and is drawn to scale, with branch lengths measured in the number of substitutions per site.

The gene order between JA11, AD1, and Y3 is highly conserved. All three genomes contain over 300 open reading frames, with each containing only between one and three unique annotated genes. These unique genes are listed in Table 5 and are all DNA or metabolism-related. There are also five genes common to AD1 and Y3 that are not found in JA11. Whilst phylogenetic clustering, as shown in Figure 7, groups AD1 and Y3 closer than Y3 and Yoloswag, it is interesting to note that the two unique genes possessed by Y3 have homologs in Yoloswag. These two phages were both isolated from apple orchards using *E. amylovora*, therefore it is surprising that they differ phylogenetically. It is possible that these unique genes are a determinant of the host range of these phages. A phylogenetic comparison of three tail fiber genes found in each genome is shown in Figure 8. This again shows a definite separation between Yoloswag and the other two phages, particularly when comparing Yoloswag_102 with Y3_104 and AD1_102, which occupy the same genomic position. This also suggests the possibility that AD1 may be capable of forming plaques on *Erwinia* species, but we have not been able to test this as we do not have access to these hosts.

**Table 5:** Unique annotated genes found in the genomes of JA11, AD, and Y3, as well as genes common to AD1 and Y3 but not present in JA11.

**Genome**	**Gene**	**Gene annotation**
JA11	JA11_30	DNA adenine methylase
AD1	AD1_017	DUF1611-domain containing protein
	AD1_258	XRE family transcriptional regulator
Y3	Y3_020	Oxygenase
	Y3_031	AntA/B antirepressor domain-containing protein
Common to AD1 and Y3	AD1_047	Transcriptional repressor
	Y3_049	
	AD1_048	DNA-cytosine methyltransferase
	Y3_050	
	AD1_018	Asparagine synthase
	Y3_018	
	AD1_267	Radical SAM superfamily protein
	Y3_272	
	AD1_016	Methyltransferase
	Y3_017	

### 2.8. Jumbo phages are capable of transduction

In a recent publication (Day et al., 2017) we tested the ability of the JA phages to facilitate transduction of chromosomal markers and plasmids between *Dickeya* cells. We can reconfirm that all of the JA jumbos are capable of transduction of a chromosomal marker between *D. solani* cells at a frequency of between 1 × 10^−6^ and 3 × 10^−4^ and report that the AD phages are capable of transduction of chromosomal markers at similar frequencies. The broader host range of the JA jumbo phages also allows transduction of plasmids between *Dickeya* species, as shown for a representative of each host range group in Table 6. JA10, the member of the *Podoviridae* family, proved incapable of transduction under the conditions tested. This makes JA10 a more promising candidate for phage therapy, and suggests the other phages may not be suitable due to the potential risk of transduction in the field.

**Table 6:** Transduction frequencies of the plasmid pECA1039-Km3 from donor *Dickeya solani* cells into other *Dickeya* species.

**Phage**	**Recipient host**	**Transduction frequency**
JA13	*Dickeya solani*	3.18 × 10^−4^
	*Dickeya dadantii* subsp. *dieffenbachiae*	5.04 × 10^−9^
	*Dickeya paradisiaca*	5.68 × 10^−5^
JA29	*Dickeya solani*	1.88 × 10^−4^
	*Dickeya dadantii* subsp. *dieffenbachiae*	6.29 × 10^−9^
JA37	*Dickeya solani*	2.31 × 10^−4^
	*Dickeya dadantii* subsp. *dieffenbachiae*	8.43 × 10^−9^
	*Dickeya paradisiaca*	7.74 × 10^−5^
	*Dickeya zeae*	4.27 × 10^−6^

*Phages are representatives of each host range group of the JA jumbo phages as detailed in Table 2*.

## 3. Discussion

All seed crops imported into Scotland, along with 10% of Scottish-origin crops, are tested for *D. solani* each year. In 2017, the most recent year for which there are data, 663 samples were tested and none were positive for *Dickeya* species (Scottish Government, 2017), which has been the finding since 2010 when rigorous testing began. Whilst there have been isolated cases of *D. solani* infection reported in England and Wales since 2007 (Cahill et al., 2010), these have all originated in crops from outside of the UK (Toth et al., 2016), therefore it is not thought that *Dickeya* is established in the UK. This begs the question as to why we have been able to isolate phages capable of infecting *D. solani* with relative ease from the River Cam. We would hypothesize that either *D. solani* is present in the environment, but has not been confirmed by testing, or there is another permissive, but currently unknown, environmental host(s) for these phage. A novel species of *Dickeya, Dickeya aquatica*, was isolated from waterways in England (Parkinson et al., 2014), and so far has only been identified in waterway environments. It is, therefore, a formal possibility that this species could be an environmental host for the phages isolated here, but this has not yet been tested.

Previously isolated phages of *D. solani* have been found almost exclusively to be members of the *Ackermannviridae* family. This has been a consistent feature of isolations spanning multiple European countries across the last decade, including from both soil and water samples, as shown in Table 1. We questioned in our recent publication whether this indicated a special relationship between *Ackermannviridae* and *D. solani* (Day et al., 2017). The phages presented here confirm that that was the result of an extrapolation from a limited viral sample set. There are at least four groups of *D. solani* phages present in waterways around Cambridge, spanning three of the four *Caudovirales* families. Phages that have been isolated on other species of *Dickeya* that are capable of forming plaques on *D. solani* have also been recently described (Alič et al., 2017b), including the fourth *Caudovirales* family, *Siphoviridae*. It is therefore apparent that we are only superficially defining the level of phage diversity present in the environment, consistent with the notion that double-stranded DNA phages alone have been predicted to outnumber their bacterial hosts by a factor of 10 to 1 (Chibani-Chennoufi et al., 2004).

All the phages presented here, apart from JA10 and AD2, appear to be representatives of a recently described “hairy *Myoviridae*” subfamily (Buttimer et al., in press). To the best of our knowledge, these are the first reported members of this subfamily isolated using *Dickeya* species. Many of the previously reported members of this subfamily were also isolated on plant-associated bacteria such as *Pseudomonas putida* (Adriaenssens et al., 2012b) and *E. amylovora* (Esplin et al., 2017; Buttimer et al., in press). Whether there is a link between this group of phages and plant-associated bacteria, or whether the recent increase in isolation of phages using phytopathogens is skewing this view remains to be determined. The proteins responsible for the “hairs” that typify this grouping remain unknown, although the identified tail fiber proteins are likely candidates for further investigation.

Infection of seed crops with *D. solani* and related species inflicts a high economic burden, and therefore there is great interest in the use of virulent (lytic) bacteriophages as potential biocontrol agents. There have been multiple tests of the stability, environmental viability and efficacy of *Dickeya* phages (Adriaenssens et al., 2012c; Alič et al., 2017b; Czajkowski et al., 2017) in which promising results have been reported. The *Dickeya* phages able to form individual plaques through productive lytic cycle replication on multiple *Dickeya* species, reported both here and by Alič et al. (2017b), are potentially more promising for use as biocontrol agents, as they would be able to act against a wider set of phytopathogens. However, we have described here, and previously (Day et al., 2017), that the majority of the *D. solani* phages (including our phages and the LIMEstone phages) are able to facilitate generalized transduction between host cells. The phages isolated by Czajkowski et al. (2014a,b, 2015a) were not reported to have been tested for generalized transduction, but, due to their classification as *Ackermannviridae* family members, and the finding that all members of this family tested to date are capable of facilitating transduction, we predict that they are likely to be capable of doing so. Alič et al. did not report testing of their phages for transduction capacity, and it is possible that they may not be transducers, but we would echo the caution of the European Medicines Agency, among others, who have stated that it is “important to ensure that therapeutic phages do not carry out generalized transduction” (Pelfrene et al., 2016). However, the results presented here do suggest that JA10, a podovirus capable of infecting three *Dickeya* species other than *D. solani*, could offer some promise as a potentially therapeutic candidate, as it has not shown transduction capabilities when tested.

Czajkowski et al. (2014a) have reported that phages D3 and D5 are capable of infecting multiple species of *Dickeya*. This conclusion was based on simple spot test assays in which undiluted spots of phage lysate were tested on bacterial top lawns and incubated, with any resultant clearing taken to show infection. It is known that applications of high titre lysates of phage to bacterial cells can cause the phenomenon of “lysis-from-without,” in which cells lyse due to membrane disruption instead of productive phage infection (Khan Mirzaei and Nilsson, 2015). Consequently, confirmation of host range requires serial dilution of the phage lysate to visualize individual plaques on a host, and this confirmatory data would be helpful when assessing the reported host range of these phages. This reasonable caution is reinforced, particularly when we consider the genome identity of nearly 100 % with other *Ackermannviridae* family members.

The phages PD10.3 and 23.1 (Czajkowski et al., 2015a) are also reported to infect both *Dickeya* and *Pectobacterium* species, although host range was determined by the same method as D3 and D5. However, adsorption and burst size data for both phages are reported on the two genera. The genomes of both phages have been sequenced and are reported as incomplete. Curiously, the largest scaffold of both is similar to the size of other *Ackermannviridae* family members (shown in Table 1) and these scaffolds share 99% identity with the full genome of LIMEstone1. The morphology of these two phages also clearly places them within the *Ackermannviridae* family. It is therefore intriguing that phages that are so similar have such different host ranges, and so further confirmatory data on the broader host range of these two phages could be biologically illuminating. Notwithstanding such observations, interpretation of *Dickeya* phage host range data should be treated with caution. We offer a salutary lesson, based on our own data, reported here, which more rigorously reinterprets previously reported host range data. Tests of the host range of our phages following the method of Czajkowski et al. have also suggested a much broader host range than we now know to be true (data not shown). Consequently, we would caution against assigning host range to phages without rigorous experimental data involving plaque formation in line with the comments of others (Khan Mirzaei and Nilsson, 2015).

## 4. Materials and methods

### 4.1. Bacterial strains, phages, culture media, and growth conditions

All bacterial strains used in this study are listed in Table 7. *Dickeya* species were routinely grown at 30°C in Luria broth (LB) or on LB agar plates (1.5%, wt/vol, agar). Phages were stored at 4°C in phage buffer (10 mM Tris-HCl, pH 7.4, 10 mM MgSO_4_, 0.01%, wt/vol, gelatine) over a few drops of NaHCO_3_ saturated chloroform.

**Table 7:** Bacterial strains and bacteriophage genomes used in this study.

**Bacterial strain**	**References**
*Dickeya chrysanthemi* NCPBB 402	Pritchard et al., 2013b
*Dickeya dadantii* subsp. *dieffenbachiae* NCPBB 2976	Pritchard et al., 2013b
*Dickeya dianthicola* NCPBB 453	Pritchard et al., 2013a
*Dickeya paradisiaca* NCPBB 2511	Pritchard et al., 2013b
*Dickeya solani* MK10	Pritchard et al., 2013a
*Dickeya zeae* NCPBB 3532	Pritchard et al., 2013b
*Dickeya solani* MK10 pECA1039-Km3	This study
**Bacteriophage genome**	**Genbank ID and references**
BF25/12	KT240186.1 (Alič et al., 2017b)
LIMEstone1	HE600015.1 (Adriaenssens et al., 2012c)
Lu11	JQ768459.1 (Adriaenssens et al., 2012b)
NCTB	LT598654.1 (Pfreundt et al., 2017)
PaBG	KF147891.1 (Sykilinda et al., 2014)
phiRSL1	AB366653 (Yamada et al., 2010)
PP74	KY084243.1 (Kabanova et al., 2018)
XF4	KY942057.1 (Day et al., 2017)
Y3	KY984068.1 (Buttimer et al., in press)
Yoloswag	KY448244.1 (Esplin et al., 2017)

### 4.2. Isolation of phages

Treated sewage effluent was collected from a sewage treatment plant in Cambridge, United Kingdom (Matilla and Salmond, 2014). River water was collected from multiple sites along the River Cam. Samples were filter sterilized using a 0.22 μm filter before 5 mL of the sample was added to 2x LB along with 500 μL of an overnight culture of *D. solani* MK10. This mixture was incubated overnight in a 250 mL flask at 30°C with shaking at 250 rpm. One milliliter of the enriched sample was mixed with 100 μL of chloroform (saturated with NaHCO_3_) and vortexed to lyse bacterial cells. After sedimentation at 16,000 × g for 4 min, 10 μL of a serial dilution series of the supernatant was mixed with 200 μL of an overnight bacterial culture and 4 mL of LB top agar. This mixture was poured as an overlay on an LBA plate and incubated overnight at 30°C. Single phage plaques were picked with a sterile toothpick, placed into 100 μL phage buffer, and shaken with 40μL of chloroform to kill any bacteria. The phages obtained were plaque purified three times. High-titer phage lysates were then obtained as described previously (Petty et al., 2006). Briefly, 10-fold serial dilutions of the phage were incubated overnight in an agar overlay. Those plates exhibiting confluent lysis (seen as a mosaic-like effect in which the plaques are close to merging) were used for lysate preparation. The top agar was removed from the plate, vortexed with chloroform before sedimentation at 2,200 × g for 20 min at 4°C. The supernatant was removed and vortexed with a few drops of chloroform to produce the final lysate.

### 4.3. Determination of host range

The host range of isolated phages was determined by plating out ten-fold serial dilutions of the phage lysates onto agar overlays containing host *Dickeya* cells and incubating overnight at 30°C. Following best practice to avoid potential confusion with “lysis from without,” only phages that produced individual plaques following serial dilution on three independent occasions were considered as being able to infect the respective host productively through a lytic cycle.

### 4.4. Transmission electron microscopy

High-titre lysates for transmission electron microscopy were obtained as described above using 0.35% (w/v) LB agarose instead of 0.35% (w/v) LB agar overlays. Ten μL of high-titre phage lysates were adsorbed onto 400-mesh copper grids with holey carbon support films (Agar Scientific, Stansted, United Kingdom) for 2 min. The copper grids were discharged in a Quorum/Emitech K100X system (Quorum, Ringmer, United Kingdom) prior to use. Excess phage suspension was removed with filter paper and phage samples were negatively stained by placing the grids for 30 s in ten μL of 2% uranyl acetate neutralized with NaOH. The grids were then blotted on filter paper to remove the excess solution and allowed to air dry. Phages were examined by transmission electron microscopy at Cambridge Advanced Imaging Centre (Department of Physiology, Development and Neuroscience, University of Cambridge) using an FEI Tecnai G2 transmission electron microscope (FEI, OR, USA). The accelerating voltage was 120.0 kV, and images were captured with an AMT XR60B digital camera running Deben software.

### 4.5. Genome sequencing and analysis

Phage genomes were sequenced on the Illumina MiSeq Sequencer at MicrobesNG (Birmingham, UK). The reads were trimmed using Trimmomatic (Bolger et al., 2014), assessed for quality using BWA-MEM (Li, 2013) and assembled using SPAdes 3.7.1 (Bankevich et al., 2012) with standard settings. Except for JA10 and AD2, all generated over 140,000 reads and had higher than 100x coverage of the full genome. JA10 generated 3,270 reads and had 26x coverage. AD2 generated 1,899 reads and had 4.79x coverage. All assembled into one contig except AD2. Annotation was performed using DNAMaster 5.23.2 (Lawrence, 2012). Genome maps were generated using Circos 0.69.6 (Krzywinski et al., 2009). Genomes were deposited in Genbank using BankIt (NCBI) and are available under accession numbers MH460459 (JA10), MH389777 (JA11), MH460460 (JA13), MH460461 (JA29), MH460462 (JA33), and MH460463 (AD1). Genomes were compared using NCBI Blast, MEGA 7.0.26 (Kumar et al., 2016) and the Artemis Comparison Tool 13.0.0 (Carver et al., 2005). Annotation tables are found in Tables S1–S6.

### 4.6. Transduction

To test for transduction, phage lysates were generated as described above on donor bacterial strains carrying the desired plasmid or chromosomal marker. All the experiments used kanamycin as the antibiotic for selection. The chromosomal marker for the JA phages was a transposon stably inserted into a protease gene. Successful transduction was therefore confirmed by a protease-negative, kanamycin-resistant phenotype in the recipient cells. The chromosomal marker for the AD phages was a transposon stably inserted into the *lacZ* gene. Successful transduction was confirmed by kanamycin-resistant recipient colonies that were white on media containing X-gal. The plasmid marker was pECA1039-Km3 and successful transduction was confirmed by plasmid extraction and gel electrophoresis under standard conditions as described previously (Day et al., 2017).

Transduction was performed by mixing phage lysate with an overnight culture of the recipient cells to achieve a multiplicity of infection of 0.01, meaning that for each phage there were one hundred bacterial cells, apart from the transduction of pECA1039-Km3 into *Dickeya dadantii* subsp. *dieffenbachiae*, which required an MOI of 0.1. The mixture was left on the lab bench at room temperature for 20 min, followed by incubation on a rotary wheel at 30°C for 30 min. The infected culture was then centrifuged and the bacterial pellets washed with LB twice to eliminate any remaining non-adsorbed phage. The bacterial pellets were resuspended in 1 mL LB and 100 μL aliquots were spread onto LBA plates with selection for the chromosomal or plasmid marker. Appropriate standard controls, which were routinely negative, were used to score for any spontaneous resistance of the recipient strain. One hundred microliters of the phage lysate was also spread onto LBA plates to confirm lysate sterility.

## Author contributions

AD, JA, and GPCS conceived and designed the experiments, analyzed the data. AD and JA performed the experiments. AD wrote the paper. AD and GPCS edited the paper.

### Conflict of interest statement

The authors declare that the research was conducted in the absence of any commercial or financial relationships that could be construed as a potential conflict of interest.
