# Supplementary material for: Jumbo Bacteriophages Are Represented Within an Increasing Diversity of Environmental Viruses Infecting the Emerging Phytopathogen, Dickeya solani
Source: Front Microbiol. 2018 Sep 12;9:2169. doi: 10.3389/fmicb.2018.02169 (PMC6143709; doi:10.3389/fmicb.2018.02169)
Supplement: Supplementary file 1 [file Data_Sheet_1.PDF]

Supplementary Material: Jumbo bacteriophages are represented within an increasing diversity of environmental viruses infecting the emerging phytopathogen, *Dickeya solani*

Andrew Day, Jiyoon Ahn and George P. C. Salmond*

*Correspondence:
Author Name: George P. C. Salmond
gpcs2@cam.ac.uk

1 SUPPLEMENTARY TABLES AND FIGURES

1.1 JA10 genome annotation table

ORF	Start	End	Annotation
1	1014	1478	putative S-adenosyl-L-methionine hydrolase,phage-associated protein
2	1480	1620	hypothetical protein
3	1680	1877	hypothetical protein
4	1890	3029	putative protein kinase
5	3102	5744	putative T3/T7-like RNA polymerase
6	5815	6060	hypothetical protein
7	6162	6338	hypothetical protein
8	6341	6604	hypothetical protein
9	6676	7713	putative DNA ligase
10	7706	7843	putative ligase
11	7897	8100	hypothetical protein
12	8097	8219	hypothetical protein
13	8257	8514	hypothetical protein
14	8511	8696	hypothetical protein
15	8693	9331	hypothetical protein
16	9324	9512	putative bacterial RNA polymerase inhibitor
17	9505	9873	hypothetical protein
18	9936	10637	putative ssDNA-binding protein
19	10637	11083	putative endonuclease
20	11085	11540	putative lysozyme
21	11612	12115	hypothetical protein
22	12320	13831	DNA primase/helicase
23	13918	14151	hypothetical protein
24	14228	14614	hypothetical protein

25	14637	16721	DNA polymerase
26	16742	17056	hypothetical protein
27	17056	17265	hypothetical protein
28	17262	17627	hypothetical protein
29	17663	18562	putative exonuclease
30	18728	18982	hypothetical protein
31	19001	19297	hypothetical protein
32	19328	19642	putative tail-assembly protein
33	19653	21257	putative head-to-tail joining protein
34	21366	22223	putative capsid and scaffold protein
35	22348	23382	putative structural protein
36	23424	23654	putative minor capsid protein
37	23728	24315	putative tail tubular protein A
38	24337	26721	putative tail tubular protein B
39	26809	27246	putative internal core protein
40	27249	27842	putative tail protein
41	27854	30118	putative tail protein
42	30144	34118	putative internal (core) protein
43	34181	35800	putative tail fibre protein
44	35803	36198	putative tail fibre assembly protein
45	36243	36452	putative holin lysis protein
46	36445	36708	putative DNA packaging protein A
47	36807	37292	putative endopeptidase
48	37292	37909	hypothetical protein
49	37925	39679	putative DNA packaging protein
50	39949	40107	hypothetical protein

Table S1: Annotation table for JA10 (Genbank reference MH460459).

1.2 JA11 genome annotation table

ORF	Start	End	Annotation
1	54	1433	putative replicative DNA helicase DnaB
2	1531	2205	Hypothetical Protein
3	2189	5002	putative terminase
4	5068	5283	Hypothetical Protein
5	5296	7239	putative portal protein
6	7239	7547	Hypothetical Protein
7	7549	7923	Hypothetical Protein
8	7971	8435	Hypothetical Protein
9	8432	9469	putative DNA polymerase I
10	9481	9930	Hypothetical Protein
11	9936	10523	putative O-acetyl-ADP-ribose deacetylase

12	10532	10846	Hypothetical Protein
13	10852	11496	putative membrane protein
14	11757	12023	putative DNA primase
15	12272	12451	putative DNA adenine methylase
16	12460	13302	Hypothetical Protein
17	13318	13461	Hypothetical Protein
18	13470	14192	Hypothetical Protein
19	14185	14439	Hypothetical Protein
20	14520	15098	Hypothetical Protein
21	15098	15463	Hypothetical Protein
22	15450	15944	putative CMP deaminase
23	15954	16406	Hypothetical Protein
24	16409	17281	Hypothetical Protein
25	17274	18377	putative thymidylate synthase
26	18419	19129	Hypothetical Protein
27	19129	19776	Hypothetical Protein
28	20125	20364	Hypothetical Protein
29	20354	20758	Hypothetical Protein
30	20804	21508	Hypothetical Protein
31	21480	21950	Hypothetical Protein
32	21943	22902	Hypothetical Protein
33	22883	23746	Hypothetical Protein
34	23796	24131	Hypothetical Protein
35	24109	24396	Hypothetical Protein
36	24751	24906	Hypothetical Protein
37	24914	25327	Hypothetical Protein
38	25324	26043	Hypothetical Protein
39	26033	26344	Hypothetical Protein
40	26341	26700	Hypothetical Protein
41	26709	27461	Hypothetical Protein
42	27463	27723	Hypothetical Protein
43	27785	28426	Hypothetical Protein
44	28546	28911	Hypothetical Protein
45	28901	29260	Hypothetical Protein
46	29271	29738	Hypothetical Protein
47	29799	30392	Hypothetical Protein
48	30395	30607	Hypothetical Protein
49	30604	30873	Hypothetical Protein
50	30881	31312	Hypothetical Protein
51	31312	31839	Hypothetical Protein
52	31814	32653	Hypothetical Protein
53	32662	32916	Hypothetical Protein
54	32913	33314	putative ASCH domain-containing protein

55	33290	34048	Hypothetical Protein
56	34035	34565	Hypothetical Protein
57	34558	34875	Hypothetical Protein
58	34862	35185	Hypothetical Protein
59	35717	35962	Hypothetical Protein
60	36000	36584	putative bifunctional (p)ppGpp synthetase/guanosine-3
61	36588	36983	Hypothetical Protein
62	37191	37652	Hypothetical Protein
63	37849	38448	Hypothetical Protein
64	38445	38810	Hypothetical Protein
65	38807	39514	Hypothetical Protein
66	39516	40238	Hypothetical Protein
67	40275	41066	Hypothetical Protein
68	41105	41572	putative membrane protein
69	41569	41850	Hypothetical Protein
70	41858	42187	putative membrane protein
71	42198	42437	Hypothetical Protein
72	42455	43087	Hypothetical Protein
73	43087	43995	Hypothetical Protein
74	44006	44560	Hypothetical Protein
75	44557	45639	Hypothetical Protein
76	45636	46229	Hypothetical Protein
77	46229	47152	Hypothetical Protein
78	47152	47619	Hypothetical Protein
79	47633	48652	Hypothetical Protein
80	48679	50253	Hypothetical Protein
81	50254	50751	putative membrane protein
82	50834	51886	Hypothetical Protein
83	52049	53425	putative T1SS secreted agglutinin RTX
84	53444	54193	Hypothetical Protein
85	54190	54681	putative membrane protein
86	54683	54958	Hypothetical Protein
87	54994	55740	Hypothetical Protein
88	55743	56540	Hypothetical Protein
89	56533	56991	hypothetical protein
90	56993	57811	putative tail fibre protein
91	57823	58023	Hypothetical Protein
92	58068	61601	putative ILEI domain-containing protein
93	61610	63631	Hypothetical Protein
94	63644	64138	putative tail fibre protein
95	64152	64784	putative tail fibre protein
96	64795	65769	putative tail protein
97	65766	68087	Hypothetical Protein

98	68175	69245	Hypothetical Protein
99	69289	73812	Hypothetical Protein
100	73809	75272	putative baseplate wedge subunit
101	75269	75682	putative baseplate protein
102	75682	75972	putative baseplate spike
103	76665	76976	Hypothetical Protein
104	77713	79491	Hypothetical Protein
105	79501	79941	Hypothetical Protein
106	79926	80519	putative dTMP kinase
107	80524	80961	putative MmcB-like DNA repair protein
108	81002	81454	putative NUDIX hydrolase
109	81451	81957	Hypothetical Protein
110	82255	82881	Hypothetical Protein
111	82894	83679	putative baseplate protein/tail-associated lysozyme
112	83676	85559	Hypothetical Protein
113	85563	85784	Hypothetical Protein
114	85791	86189	Hypothetical Protein
115	86189	86482	Hypothetical Protein
116	86455	87258	Hypothetical Protein
117	87258	90050	putative VgrG-like protein/endolysin
118	90050	90892	Hypothetical Protein
119	90885	91571	Hypothetical Protein
120	91582	92094	putative tail tube protein
121	92097	92765	Hypothetical Protein
122	92808	93323	putative tail tube protein
123	93338	95023	putative tail sheath protein
124	95081	95425	Hypothetical Protein
125	95427	96089	Hypothetical Protein
126	96161	96634	Hypothetical Protein
127	96726	97820	putative major capsid protein
128	97873	98577	putative structural protein
129	98647	100605	putative ATPase
130	100689	101792	Hypothetical Protein
131	101789	102610	putative prohead core protein protease
132	102617	103039	Hypothetical Protein
133	103044	103931	Hypothetical Protein
134	103939	105039	putative glycosyl transferase
135	105050	105964	Hypothetical Protein
136	105964	107934	putative DNA ligase
137	107958	108881	Hypothetical Protein
138	108878	109720	Hypothetical Protein
139	109720	111651	Hypothetical Protein
140	111711	115673	Hypothetical Protein

141	115735	116889	Hypothetical Protein
142	116908	117882	Hypothetical Protein
143	117886	118650	Hypothetical Protein
144	118666	121749	putative major tail protein/T1SS secreted agglutinin RTX
145	121739	122383	Hypothetical Protein
146	122380	123006	Hypothetical Protein
147	123028	124704	putative tail sheath protein
148	124792	125838	Hypothetical Protein
149	125826	126680	Hypothetical Protein
150	126685	126894	Hypothetical Protein
151	126891	127523	Hypothetical Protein
152	127560	127841	Hypothetical Protein
153	127858	128241	Hypothetical Protein
154	128207	128545	Hypothetical Protein
155	128551	129444	putative DNA repair helicase
156	130521	131657	putative DNA repair helicase
157	131660	132223	Hypothetical Protein
158	132225	132653	Hypothetical Protein
159	132650	133825	Hypothetical Protein
160	133812	134081	Hypothetical Protein
161	134078	137233	putative DNA polymerase I
162	137328	137645	Hypothetical Protein
163	137648	138202	Hypothetical Protein
164	138246	144080	putative ATP-dependent DNA helicase
165	144091	144591	Hypothetical Protein
166	144604	145371	Hypothetical Protein
167	145371	145730	putative HNH family endonuclease
168	145737	146495	Hypothetical Protein
169	146556	147359	Hypothetical Protein
170	147412	148572	putative head to tail joining protein
171	148575	149672	Hypothetical Protein
172	149672	150949	Hypothetical Protein
173	151090	151752	Hypothetical Protein
174	151888	153018	putative recombination-related endonuclease
175	153133	153657	putative ssDNA binding protein
176	153704	155668	Hypothetical Protein
177	155665	157452	Hypothetical Protein
178	157463	157828	putative DUF2778 domain-containing protein
179	157828	158394	Hypothetical Protein
180	158413	159405	Hypothetical Protein
181	159408	159875	Hypothetical Protein
182	159872	160447	putative glycosyl hydrolase
183	160527	160850	Hypothetical Protein

184	160850	161563	Hypothetical Protein
185	161566	161817	Hypothetical Protein
186	161817	164117	putative exonuclease
187	164120	164320	Hypothetical Protein
188	164401	164637	Hypothetical Protein
189	164654	165088	Hypothetical Protein
190	165091	165768	Hypothetical Protein
191	165889	166368	Hypothetical Protein
192	166448	167668	putative DNA polymerase III
193	168011	168586	Hypothetical Protein
194	168586	168918	Hypothetical Protein
195	168908	169885	Hypothetical Protein
196	169928	170395	Hypothetical Protein
197	170402	170896	Hypothetical Protein
198	170908	171372	Hypothetical Protein
199	171421	172509	Hypothetical Protein
200	172547	173764	Hypothetical Protein
201	174627	175178	Hypothetical Protein
202	175181	176299	Hypothetical Protein
203	176443	176991	putative holliday junction resolvase
204	177004	177645	Hypothetical Protein
205	177704	178342	Hypothetical Protein
206	178339	180324	putative inverse autotransporter beta-barrel domain-containing protein
207	180446	181123	Hypothetical Protein
208	181159	182214	putative DNA primase
209	182280	182615	Hypothetical Protein
210	182653	183669	putative exonuclease
211	183677	184078	Hypothetical Protein
212	184532	185194	Hypothetical Protein
213	185197	185361	Hypothetical Protein
214	185373	185645	Hypothetical Protein
215	185642	185959	Hypothetical Protein
216	186002	186178	Hypothetical Protein
217	186181	186702	Hypothetical Protein
218	186699	187148	putative cyclic phosphodiesterase
219	187132	187413	Hypothetical Protein
220	187449	188486	Hypothetical Protein
221	188547	189752	putative ssDNA binding protein
222	189807	191312	putative RecA protein
223	191354	191755	Hypothetical Protein
224	191872	192651	Hypothetical Protein
225	192648	193226	Hypothetical Protein
226	193210	193743	Hypothetical Protein

227	193824	194072	Hypothetical Protein
228	194136	195953	Hypothetical Protein
229	196006	196599	Hypothetical Protein
230	196538	197179	Hypothetical Protein
231	197227	197868	Hypothetical Protein
232	197868	198185	Hypothetical Protein
233	198163	199332	putative methyltransferase
234	199388	199534	Hypothetical Protein
235	199513	200736	putative DNA adenine methylase
236	200913	201248	Hypothetical Protein
237	201311	201790	Hypothetical Protein
238	201790	202305	Hypothetical Protein
239	202295	202720	Hypothetical Protein
240	202689	202964	Hypothetical Protein
241	202964	203182	Hypothetical Protein
242	203184	203942	Hypothetical Protein
243	204209	204802	Hypothetical Protein
244	204786	205136	Hypothetical Protein
245	205145	205495	Hypothetical Protein
246	205507	205755	Hypothetical Protein
247	205926	206198	Hypothetical Protein
248	206263	206691	Hypothetical Protein
249	206751	207098	Hypothetical Protein
250	207095	207388	Hypothetical Protein
251	207398	208183	Hypothetical Protein
252	208180	208788	Hypothetical Protein
253	208785	209504	Hypothetical Protein
254	209506	210186	Hypothetical Protein
255	210235	210906	Hypothetical Protein
256	210903	211121	Hypothetical protein
257	211543	212043	Hypothetical Protein
258	212045	212761	Hypothetical Protein
259	212761	213003	Hypothetical Protein
260	213123	213461	Hypothetical Protein
261	213464	214084	Hypothetical Protein
262	214081	214623	putative RNA 2'-phosphotransferase
263	214726	215118	Hypothetical Protein
264	215128	215385	putative DksA/TraR family C4-type zinc finger protein
265	215388	215696	Hypothetical Protein
266	215699	215839	Hypothetical Protein
267	215839	216273	Hypothetical Protein
268	216292	216741	Hypothetical Protein
269	216777	217712	putative UvsE UV damage repair endonuclease

270	217703	217804	Hypothetical Protein
271	217813	218622	Hypothetical Protein
272	218633	219241	Hypothetical Protein
273	219242	221191	Hypothetical Protein
274	221244	221903	Hypothetical Protein
275	222039	222218	Hypothetical Protein
276	222222	222470	Hypothetical Protein
277	222474	222932	Hypothetical Protein
278	222935	223231	Hypothetical Protein
279	223244	223537	Hypothetical Protein
280	223592	224035	Hypothetical Protein
281	224038	224328	Hypothetical Protein
282	224328	224894	putative dUTPase
283	225272	225790	putative lytic transglycosylase
284	225937	226230	Hypothetical Protein
285	226246	226758	Hypothetical Protein
286	226771	226914	Hypothetical Protein
287	226957	227664	Hypothetical Protein
288	227664	228188	Hypothetical Protein
289	228185	228640	Hypothetical Protein
290	228641	228847	Hypothetical Protein
291	228854	229258	Hypothetical Protein
292	229309	229932	Hypothetical Protein
293	230033	230638	Hypothetical Protein
294	230635	230811	Hypothetical Protein
295	230808	231215	Hypothetical Protein
296	231226	232059	Hypothetical Protein
297	232076	232858	Hypothetical Protein
298	233013	233363	Hypothetical Protein
299	233514	234257	Hypothetical Protein
300	234313	234843	Hypothetical Protein
301	234850	235227	Hypothetical Protein
302	235227	235592	Hypothetical Protein
303	235878	237491	Hypothetical Protein
304	237564	238490	Hypothetical Protein
305	238490	238852	Hypothetical Protein
306	238861	239337	Hypothetical Protein
307	239309	240577	Hypothetical Protein
308	240577	243549	Hypothetical Protein
309	243604	244356	Hypothetical Protein
310	244379	244957	Hypothetical Protein
311	245162	245953	Hypothetical Protein
312	245985	246080	Hypothetical Protein

313	246258	246776	Hypothetical Protein
314	246870	247211	Hypothetical Protein
315	247330	249390	putative DNA topoisomerase IV/gyrase subunit B
316	249390	251072	putative DNA topoisomerase 4 subunit A
317	251226	252416	Hypothetical Protein
318	252406	252948	Hypothetical Protein
319	252920	253759	Hypothetical protein
320	253821	254954	Hypothetical Protein
321	254962	255318	Hypothetical Protein

Table S2: Annotation table for JA11 (Genbank reference MH389777).

1.3 JA13 genome annotation table

ORF	Start	End	Annotation
1	74	1453	putative replicative DNA helicase DnaB
2	1446	2225	hypothetical protein
3	2209	5022	putative terminase
4	5215	5430	Hypothetical protein
5	5443	7386	putative portal protein
6	7386	7694	hypothetical protein
7	7696	8070	hypothetical protein
8	8118	8582	hypothetical protein
9	8579	9616	putative DNA polymerase I
10	9628	10077	hypothetical protein
11	10083	10670	putative O-acetyl-ADP-ribose deacetylase
12	10679	10993	hypothetical protein
13	10999	11643	putative membrane protein
14	11904	12170	putative DNA primase
15	12193	12414	hypothetical protein
16	12419	12598	hypothetical protein
17	12607	13449	putative DNA adenine methylase
18	13465	13608	hypothetical protein
19	13617	14339	hypothetical protein
20	14332	14586	hypothetical protein
21	14667	15245	hypothetical protein
22	15245	15610	hypothetical protein
23	15597	16091	CMP deaminase
24	16101	16553	hypothetical protein
25	16556	17428	hypothetical protein
26	17421	18524	thymidylate synthase
27	18566	19276	hypothetical protein
28	19276	19923	hypothetical protein

29	20272	20511	hypothetical protein
30	20501	20905	hypothetical protein
31	20951	21655	hypothetical protein
32	21627	22097	hypothetical protein
33	22090	23049	hypothetical protein
34	23030	23893	hypothetical protein
35	23943	24278	hypothetical protein
36	24256	24543	hypothetical protein
37	24644	24895	hypothetical protein
38	24898	25053	hypothetical protein
39	25061	25474	hypothetical protein
40	25471	26190	hypothetical protein
41	26180	26491	hypothetical protein
42	26488	26844	hypothetical protein
43	26853	27605	hypothetical protein
44	27607	27867	hypothetical protein
45	27930	28571	hypothetical protein
46	28691	29056	hypothetical protein
47	29046	29405	hypothetical protein
48	29416	29883	hypothetical protein
49	29944	30537	hypothetical protein
50	30540	30752	hypothetical protein
51	30749	31018	hypothetical protein
52	31027	31488	hypothetical protein
53	31463	32302	hypothetical protein
54	32311	32565	hypothetical protein
55	32562	32963	putative ASCH domain-containing protein
56	32939	33697	hypothetical protein
57	33684	34214	hypothetical protein
58	34207	34524	hypothetical protein
59	34511	34834	hypothetical protein
60	35155	36198	hypothetical protein
61	36195	36779	putative bifunctional (p)ppGpp synthetase/guanosine-3
62	36783	37178	hypothetical protein
63	37386	37847	hypothetical protein
64	38044	38643	hypothetical protein
65	38640	39005	hypothetical protein
66	39002	39709	hypothetical protein
67	39711	40433	hypothetical protein
68	40470	41261	hypothetical protein
69	41300	41767	putative membrane protein
70	41764	42045	hypothetical protein
71	42053	42382	putative membrane protein

72	42393	42632	hypothetical protein
73	42650	43282	hypothetical protein
74	43282	44190	hypothetical protein
75	44201	44755	hypothetical protein
76	44752	45834	hypothetical protein
77	45831	46424	hypothetical protein
78	46424	47347	hypothetical protein
79	47347	47814	hypothetical protein
80	47828	48847	hypothetical protein
81	48874	50448	hypothetical protein
82	50449	50946	putative membrane protein
83	51029	52081	putative T1SS secreted agglutinin RTX
84	52244	53620	hypothetical protein
85	53639	54388	putative membrane protein
86	54385	54876	hypothetical protein
87	54878	55153	hypothetical protein
88	55189	55935	hypothetical protein
89	55938	56735	hypothetical protein
90	56728	57186	hypothetical protein
91	57188	58006	putative tail fibre protein
92	58018	58218	Hypothetical protein
93	58263	61796	putative ILEI domain-containing protein
94	61805	63826	hypothetical protein
95	63839	64333	putative tail fibre protein
96	64347	64979	putative tail fibre protein
97	64990	65964	putative tail protein
98	65961	68282	hypothetical protein
99	68370	69440	hypothetical protein
100	69484	74007	hypothetical protein
101	74004	75467	putative baseplate wedge subunit protein
102	75464	75877	putative baseplate protein
103	75877	76167	putative baseplate spike protein
104	76860	77171	hypothetical protein
105	77908	79686	hypothetical protein
106	79696	80136	hypothetical protein
107	80121	80714	putative dTMP kinase
108	80719	81156	putative MmcB-like DNA repair protein
109	81197	81649	putative NUDIX hydrolase
110	81646	82152	hypothetical protein
111	82450	83076	hypothetical protein
112	83089	83871	putative baseplate protein/tail-associated lysozyme
113	83871	85754	hypothetical protein
114	85758	85979	hypothetical protein

115	85986	86384	hypothetical protein
116	86384	86677	hypothetical protein
117	86650	87453	hypothetical protein
118	87453	90245	putative VgrG-like protein/endolysin
119	90245	91087	hypothetical protein
120	91080	91766	hypothetical protein
121	91777	92289	putative tail tube protein
122	92292	92960	hypothetical protein
123	93003	93518	putative tail tube protein
124	93533	95218	putative tail sheath protein
125	95276	95620	hypothetical protein
126	95622	96284	hypothetical protein
127	96355	96828	hypothetical protein
128	96920	98014	putative major capsid protein
129	98067	98771	putative structural protein
130	98841	100799	putative ATPase
131	100883	101929	hypothetical protein
132	101986	102804	putative prohead core protein protease
133	102811	103233	hypothetical protein
134	103238	104125	hypothetical protein
135	104133	105233	putative glycosyl transferase
136	105244	106158	hypothetical protein
137	106158	108128	putative DNA ligase
138	108152	109075	hypothetical protein
139	109072	109914	hypothetical protein
140	109914	111878	hypothetical protein
141	111938	115900	hypothetical protein
142	115962	117116	hypothetical protein
143	117135	118109	hypothetical protein
144	118113	118877	hypothetical protein
145	118893	121976	putative major tail protein/T1SS secreted agglutinin RTX
146	121966	122610	hypothetical protein
147	122607	123233	hypothetical protein
148	123255	124931	putative tail sheath protein
149	125019	126065	hypothetical protein
150	126053	126907	hypothetical protein
151	126912	127121	hypothetical protein
152	127118	127750	hypothetical protein
153	127787	128068	hypothetical protein
154	128085	128468	hypothetical protein
155	128434	128772	hypothetical protein
156	128778	130388	putative DNA repair helicase
157	130391	130954	hypothetical protein

158	130956	131384	hypothetical protein
159	131381	132556	hypothetical protein
160	132543	132812	hypothetical protein
161	132809	135964	putative DNA polymerase I
162	136060	136377	hypothetical protein
163	136380	136934	hypothetical protein
164	136978	142812	putative ATP-dependent DNA helicase
165	142823	143323	hypothetical protein
166	143336	144103	hypothetical protein
167	144103	144462	putative HNH family endonuclease
168	144469	145227	hypothetical protein
169	145288	146091	hypothetical protein
170	146144	147304	putative head to tail joining protein
171	147307	148404	hypothetical protein
172	148404	149681	hypothetical protein
173	149822	150484	hypothetical protein
174	150620	151750	putative recombination-related endonuclease
175	151865	152389	putative ssDNA binding protein
176	152436	154400	hypothetical protein
177	154397	156184	hypothetical protein
178	156195	156560	putative DUF2778 domain-containing protein
179	156560	157126	hypothetical protein
180	157145	158173	hypothetical protein
181	158176	158643	hypothetical protein
182	158640	159215	putative glycosyl hydrolase
183	159295	159618	hypothetical protein
184	159618	160331	hypothetical protein
185	160334	160585	hypothetical protein
186	160585	162885	putative exonuclease
187	162888	163088	hypothetical protein
188	163169	163405	hypothetical protein
189	163422	163856	hypothetical protein
190	163859	164536	hypothetical protein
191	164657	165136	hypothetical protein
192	165216	166436	putative DNA polymerase III
193	166779	167354	hypothetical protein
194	167354	167686	hypothetical protein
195	167676	168653	hypothetical protein
196	168696	169163	hypothetical protein
197	169170	169664	hypothetical protein
198	169676	170140	hypothetical protein
199	170189	171328	hypothetical protein
200	171366	172220	hypothetical protein

201	173464	174015	hypothetical protein
202	174018	175136	hypothetical protein
203	175280	175828	putative holliday junction resolvase
204	175841	176536	hypothetical protein
205	176598	177215	hypothetical protein
206	177212	179197	putative inverse autotransporter beta-barrel domain-containing protein
207	179319	179996	hypothetical protein
208	180032	181087	putative DNA primase
209	181153	181488	hypothetical protein
210	181526	182542	putative exonuclease
211	182550	182951	hypothetical protein
212	182902	183489	hypothetical protein
213	183405	184067	hypothetical protein
214	184070	184234	hypothetical protein
215	184246	184518	hypothetical protein
216	184515	184832	hypothetical protein
217	184875	185051	hypothetical protein
218	185054	185575	putative cyclic phosphodiesterase
219	185572	186021	hypothetical protein
220	186005	186286	hypothetical protein
221	186322	187359	hypothetical protein
222	187420	188607	putative ssDNA binding protein
223	188662	190167	putative RecA recombinase
224	190209	190610	hypothetical protein
225	190728	191507	hypothetical protein
226	191504	192082	hypothetical protein
227	192066	192599	hypothetical protein
228	192680	192928	hypothetical protein
229	192992	194809	hypothetical protein
230	194862	195455	hypothetical protein
231	195403	196035	hypothetical protein
232	196035	196724	hypothetical protein
233	196724	197041	hypothetical protein
234	197019	198188	putative methyltransferase
235	198202	198390	hypothetical protein
236	198369	199592	putative DNA adenine methylase
237	199769	200104	hypothetical protein
238	200167	200646	hypothetical protein
239	200646	201161	hypothetical protein
240	201151	201576	hypothetical protein
241	201545	201820	hypothetical protein
242	201820	202038	hypothetical protein
243	202040	202798	hypothetical protein

244	203065	203658	hypothetical protein
245	203642	203992	hypothetical protein
246	204001	204351	hypothetical protein
247	204363	204611	hypothetical protein
248	204782	205054	hypothetical protein
249	205119	205547	hypothetical protein
250	205608	205955	hypothetical protein
251	205952	206245	hypothetical protein
252	206255	207040	hypothetical protein
253	207037	207645	hypothetical protein
254	207642	208361	hypothetical protein
255	208361	209023	Hypothetical protein
256	209094	209765	hypothetical protein
257	209762	209980	Hypothetical protein
258	209989	210198	hypothetical protein
259	210201	210407	hypothetical protein
260	210404	210904	hypothetical protein
261	210906	211622	hypothetical protein
262	211622	211864	hypothetical protein
263	211984	212322	hypothetical protein
264	212325	212945	hypothetical protein
265	212942	213484	putative RNA 2'-phosphotransferase
266	213587	213979	hypothetical protein
267	213989	214246	putative DksA/TraR family C4-type zinc finger protein
268	214249	214557	hypothetical protein
269	214560	214700	hypothetical protein
270	214700	215134	hypothetical protein
271	215154	215603	hypothetical protein
272	215639	216574	putative UvsE UV damage endonuclease
273	216565	216666	hypothetical protein
274	216675	217484	hypothetical protein
275	217492	218103	hypothetical protein
276	218104	220080	hypothetical protein
277	220133	220792	hypothetical protein
278	220928	221107	hypothetical protein
279	221111	221359	hypothetical protein
280	221363	221821	hypothetical protein
281	221824	222120	hypothetical protein
282	222133	222426	hypothetical protein
283	222481	222924	hypothetical protein
284	222927	223217	hypothetical protein
285	223217	223783	putative dUTPase
286	224161	224679	putative lytic transglycosylase

287	224826	225119	hypothetical protein
288	225135	225647	hypothetical protein
289	225660	225803	hypothetical protein
290	225846	226553	Hypothetical protein
291	226553	227077	hypothetical protein
292	227074	227529	hypothetical protein
293	227530	227736	hypothetical protein
294	227743	228147	hypothetical protein
295	228196	228819	hypothetical protein
296	228920	229525	hypothetical protein
297	229522	229698	hypothetical protein
298	229695	230102	hypothetical protein
299	230113	230946	hypothetical protein
300	230963	231745	hypothetical protein
301	231900	232250	hypothetical protein
302	232401	233144	hypothetical protein
303	233200	233730	hypothetical protein
304	233736	234113	hypothetical protein
305	234113	234478	hypothetical protein
306	234763	236376	hypothetical protein
307	236449	237375	hypothetical protein
308	237375	237737	hypothetical protein
309	237746	238222	hypothetical protein
310	238194	239462	hypothetical protein
311	239462	242410	hypothetical protein
312	242465	243217	hypothetical protein
313	243240	243818	hypothetical protein
314	244023	244805	hypothetical protein
315	244983	245501	hypothetical protein
316	245595	245936	hypothetical protein
317	246055	248115	putative DNA topoisomerase IV/gyrase subunit B
318	248115	249797	putative DNA topoisomerase 4 subunit A
319	249951	251141	hypothetical protein
320	251131	251673	hypothetical protein
321	251660	252484	Hypothetical protein
322	252546	253679	hypothetical protein
323	253687	254043	hypothetical protein

Table S3: Annotation table for JA13 (Genbank reference MH460460).

1.4 JA29 genome annotation table

ORF	Start	End	Annotation

1	4	1467	putative replicative DNA helicase DnaB
2	1565	2239	hypothetical protein
3	2223	5036	putative terminase
4	5100	5315	hypothetical protein
5	5328	7271	putative portal protein
6	7271	7579	hypothetical protein
7	7581	7955	hypothetical protein
8	8002	8463	hypothetical protein
9	8460	9497	putative DNA polymerase I
10	9509	9958	hypothetical protein
11	9964	10548	putative O-acetyl-ADP-ribose deacetylase
12	10564	10878	hypothetical protein
13	10884	11528	putative membrane protein
14	11789	12055	putative DNA primase
15	12226	12483	hypothetical protein
16	12492	13334	putative DNA adenine methylase
17	13348	13491	hypothetical protein
18	13499	14218	hypothetical protein
19	14211	14444	hypothetical protein
20	14518	15096	hypothetical protein
21	15135	15461	hypothetical protein
22	15448	15942	putative CMP deaminase
23	15952	16404	hypothetical protein
24	16407	17282	hypothetical protein
25	17275	18039	putative thymidylate synthase
26	18173	18973	putative GIY-YIG family homing endonuclease
27	19151	19504	putative thymidylate synthase
28	19545	20270	hypothetical protein
29	20270	20917	hypothetical protein
30	21259	21498	hypothetical protein
31	21488	21892	hypothetical protein
32	21938	22642	hypothetical protein
33	22614	23084	hypothetical protein
34	23077	24042	hypothetical protein
35	24023	24886	hypothetical protein
36	24936	25271	hypothetical protein
37	25249	25536	hypothetical protein
38	25577	25888	hypothetical protein
39	25891	26046	hypothetical protein
40	26054	26467	hypothetical protein
41	26464	27183	hypothetical protein
42	27173	27484	hypothetical protein
43	27481	27840	hypothetical protein

44	27849	28601	hypothetical protein
45	28603	28863	hypothetical protein
46	28924	29565	hypothetical protein
47	29688	30053	hypothetical protein
48	30043	30402	hypothetical protein
49	30413	30880	hypothetical protein
50	30942	31532	hypothetical protein
51	31535	31756	hypothetical protein
52	31753	32022	hypothetical protein
53	32031	32462	hypothetical protein
54	32470	33309	hypothetical protein
55	33321	33722	putative ASCH domain-containing protein
56	33698	34531	hypothetical protein
57	34518	35048	hypothetical protein
58	35041	35361	hypothetical protein
59	35348	35671	hypothetical protein
60	35691	36032	hypothetical protein
61	36200	36445	putative bifunctional (p)ppGpp synthetase/guanosine-3’ 5’-bis diphosphate 3’-pyrophosphohydrolase protein
62	36483	37067	hypothetical protein
63	37071	37466	hypothetical protein
64	37435	37665	hypothetical protein
65	37673	38134	hypothetical protein
66	38328	38927	hypothetical protein
67	38924	39289	hypothetical protein
68	39286	39996	hypothetical protein
69	39998	40702	hypothetical protein
70	40737	41492	hypothetical protein
71	41531	41998	putative membrane protein
72	41995	42276	hypothetical protein
73	42284	42613	putative membrane protein
74	42624	42863	hypothetical protein
75	42881	43513	hypothetical protein
76	43513	44421	hypothetical protein
77	44432	44986	hypothetical protein
78	44983	46065	hypothetical protein
79	46062	46658	hypothetical protein
80	46658	47581	hypothetical protein
81	47581	48048	hypothetical protein
82	48062	49081	hypothetical protein
83	49108	50682	hypothetical protein
84	50683	51180	putative membrane protein
85	51263	52315	hypothetical protein

86	52478	53854	putative T1SS secreted agglutinin RTX
87	53873	54622	hypothetical protein
88	54619	55110	putative membrane protein
89	55112	55387	hypothetical protein
90	55424	56170	hypothetical protein
91	56173	56970	hypothetical protein
92	56963	57421	hypothetical protein
93	57423	58241	putative tail fibre protein
94	58253	58453	hypothetical protein
95	58498	62031	putative ILEI domain-containing protein
96	62040	64061	hypothetical protein
97	64074	64568	putative tail fibre protein
98	64582	65214	putative tail fibre protein
99	65225	66199	putative tail protein
100	66196	68517	hypothetical protein
101	68604	69674	hypothetical protein
102	69720	74243	hypothetical protein
103	74240	75703	putative baseplate wedge subunit protein
104	75700	76113	putative baseplate protein
105	76113	76403	putative baseplate spike protein
106	77095	77406	hypothetical protein
107	78410	80188	hypothetical protein
108	80198	80638	hypothetical protein
109	80623	81216	putative dTMP kinase
110	81221	81709	putative MmcB-like DNA repair protein
111	81699	82151	putative NUDIX hydrolase
112	82148	82654	hypothetical protein
113	82952	83578	hypothetical protein
114	83588	84376	putative baseplate protein/tail-associated lysozyme
115	84373	86256	hypothetical protein
116	86260	86481	hypothetical protein
117	86488	86874	hypothetical protein
118	86874	87167	hypothetical protein
119	87140	87943	hypothetical protein
120	87943	90741	putative VgrG-like protein/endolysin
121	90741	91583	hypothetical protein
122	91576	92262	hypothetical protein
123	92273	92785	putative tail tube protein
124	92788	93456	hypothetical protein
125	93499	94014	putative tail tube protein
126	94029	95714	putative tail sheath protein
127	95772	96116	hypothetical protein
128	96118	96780	hypothetical protein

129	96851	97324	hypothetical protein
130	97420	98514	putative major capsid protein
131	98568	99272	putative structural protein
132	99337	101325	putative ATPase
133	101409	102512	hypothetical protein
134	102512	103330	putative prohead core protease
135	103337	103759	hypothetical protein
136	103764	104651	hypothetical protein
137	104659	105759	putative glycosyl transferase
138	105770	106684	hypothetical protein
139	106684	108654	putative DNA ligase
140	108678	109601	hypothetical protein
141	109598	110440	hypothetical protein
142	110440	112365	hypothetical protein
143	112425	116387	hypothetical protein
144	116451	117605	hypothetical protein
145	117624	118598	hypothetical protein
146	118602	119366	hypothetical protein
147	119382	122465	putative major tail protein/T1SS secreted agglutinin RTX
148	122455	123099	hypothetical protein
149	123096	123722	hypothetical protein
150	123744	125420	putative tail sheath protein
151	125508	126554	hypothetical protein
152	126542	127396	hypothetical protein
153	127401	127610	hypothetical protein
154	127607	128239	hypothetical protein
155	128275	128556	hypothetical protein
156	128573	128956	hypothetical protein
157	128922	129260	hypothetical protein
158	129266	130876	putative DNA repair helicase
159	130879	131439	hypothetical protein
160	131441	131869	hypothetical protein
161	131866	133041	hypothetical protein
162	133028	133297	hypothetical protein
163	133294	136449	putative DNA polymerase I
164	136544	136861	hypothetical protein
165	136864	137418	hypothetical protein
166	137462	143296	putative ATP-dependent DNA helicase
167	143307	143807	hypothetical protein
168	143820	144587	hypothetical protein
169	144587	144946	putative HNH family endonuclease
170	144953	145717	hypothetical protein
171	145777	146580	hypothetical protein

172	146633	147793	putative head to tail joining protein
173	147796	148893	hypothetical protein
174	148893	150167	hypothetical protein
175	150308	150973	hypothetical protein
176	151109	152239	putative recombination-related endonuclease
177	152353	152754	putative ssDNA binding protein
178	152924	154885	hypothetical protein
179	154882	156669	hypothetical protein
180	156680	157045	putative DUF2778 domain-containing protein
181	157045	157611	hypothetical protein
182	157630	158691	hypothetical protein
183	158695	159162	hypothetical protein
184	159159	159734	putative glycosyl hydrolase
185	159814	160137	hypothetical protein
186	160137	160850	hypothetical protein
187	160841	161104	hypothetical protein
188	161104	163404	putative exonuclease
189	163407	163607	hypothetical protein
190	163692	163925	hypothetical protein
191	163925	164359	hypothetical protein
192	164362	165039	hypothetical protein
193	165159	165638	hypothetical protein
194	165718	166938	putative DNA polymerase III
195	167276	167851	hypothetical protein
196	167851	168183	hypothetical protein
197	168173	169150	hypothetical protein
198	169195	169671	hypothetical protein
199	169679	170173	hypothetical protein
200	170185	170406	hypothetical protein
201	170696	171835	hypothetical protein
202	171873	173510	hypothetical protein
203	173970	174521	hypothetical protein
204	174524	175642	hypothetical protein
205	175786	176334	putative holliday junction resolvase
206	176347	176988	hypothetical protein
207	177047	177685	hypothetical protein
208	177682	179667	putative autotransporter beta-barrel domain-containing protein
209	179789	180469	hypothetical protein
210	180503	181567	putative DNA primase
211	181633	181968	hypothetical protein
212	182006	183022	putative exonuclease
213	183030	183431	hypothetical protein
214	183885	184547	hypothetical protein

215	184550	184714	hypothetical protein
216	184714	184998	hypothetical protein
217	184995	185318	hypothetical protein
218	185362	185538	hypothetical protein
219	185541	186059	hypothetical protein
220	186056	186505	putative cyclic phosphodiesterase
221	186489	186770	hypothetical protein
222	186806	187843	hypothetical protein
223	187904	189088	putative ssDNA binding protein
224	189143	190648	putative RecA protein
225	190690	191091	hypothetical protein
226	191209	191988	hypothetical protein
227	191985	192563	hypothetical protein
228	192547	193080	hypothetical protein
229	193161	193409	hypothetical protein
230	193475	195292	hypothetical protein
231	195345	195938	hypothetical protein
232	195877	196518	hypothetical protein
233	196566	197207	hypothetical protein
234	197207	197524	hypothetical protein
235	197502	198671	hypothetical protein
236	198685	198885	putative methyltransferase
237	198864	200087	putative DNA adenine methylase
238	200265	200600	hypothetical protein
239	200661	201140	hypothetical protein
240	201140	201655	hypothetical protein
241	201645	202070	hypothetical protein
242	202117	202314	hypothetical protein
243	202314	202532	hypothetical protein
244	202535	203293	hypothetical protein
245	203560	204153	hypothetical protein
246	204137	204487	hypothetical protein
247	204496	204846	hypothetical protein
248	204857	205105	hypothetical protein
249	205274	205546	hypothetical protein
250	205608	206021	hypothetical protein
251	206085	206429	hypothetical protein
252	206511	206924	hypothetical protein
253	206970	207260	hypothetical protein
254	207270	208061	hypothetical protein
255	208058	208375	hypothetical protein
256	208490	209161	hypothetical protein
257	209158	209376	Hypothetical protein

258	209798	210298	hypothetical protein
259	210300	211016	hypothetical protein
260	211016	211258	hypothetical protein
261	211270	211716	hypothetical protein
262	211719	212339	hypothetical protein
263	212336	212878	putative RNA 2'-phosphotransferase
264	212983	213813	hypothetical protein
265	213855	214112	putative DksA/TraR family C4-type zinc finger protein
266	214115	214252	hypothetical protein
267	214252	214674	hypothetical protein
268	214692	215102	hypothetical protein
269	215163	216098	putative UvsE UV damage endonuclease
270	216089	216190	hypothetical protein
271	216199	217011	hypothetical protein
272	217019	217630	hypothetical protein
273	217631	219796	hypothetical protein
274	219847	220512	hypothetical protein
275	220661	220840	hypothetical protein
276	220844	221092	hypothetical protein
277	221096	221554	hypothetical protein
278	221557	221853	hypothetical protein
279	221863	222150	hypothetical protein
280	222205	222645	hypothetical protein
281	222681	222974	hypothetical protein
282	222977	223543	putative dUTPase
283	223921	224439	putative lytic transglycosylase
284	224587	224883	hypothetical protein
285	224895	225407	hypothetical protein
286	225606	226313	hypothetical protein
287	226313	226840	hypothetical protein
288	226837	227289	hypothetical protein
289	227290	227496	hypothetical protein
290	227503	227907	hypothetical protein
291	227955	228578	hypothetical protein
292	228678	229283	hypothetical protein
293	229280	229456	hypothetical protein
294	229453	229860	hypothetical protein
295	229871	230704	hypothetical protein
296	230722	231504	hypothetical protein
297	231658	232008	hypothetical protein
298	232158	232895	hypothetical protein
299	232963	233343	hypothetical protein
300	233343	233708	hypothetical protein

301	233996	235612	hypothetical protein
302	235685	236614	hypothetical protein
303	236614	236988	hypothetical protein
304	236985	237461	hypothetical protein
305	237433	238701	hypothetical protein
306	238701	241661	hypothetical protein
307	241717	242469	hypothetical protein
308	242492	243067	hypothetical protein
309	243273	244073	hypothetical protein
310	244245	244766	hypothetical protein
311	244859	245200	hypothetical protein
312	245320	247395	putative DNA topoisomerase IV/gyrase subunit B
313	247395	249077	putative DNA topoisomerase 4 subunit A
314	249227	250417	hypothetical protein
315	250407	250949	hypothetical protein
316	250921	251760	Hypothetical protein
317	251822	252955	hypothetical protein
318	252963	253319	hypothetical protein

Table S4: Annotation table for JA29 (Genbank reference MH460461).

1.5 JA33 genome annotation table

ORF	Start	End	Annotation
1	44	1423	putative replicative DNA helicase DnaB
2	1521	2195	Hypothetical Protein
3	2179	4992	putative terminase
4	5058	5273	Hypothetical Protein
5	5286	7229	putative portal protein
6	7229	7537	Hypothetical Protein
7	7539	7913	Hypothetical Protein
8	7961	8425	Hypothetical Protein
9	8422	9459	putative DNA polymerase I
10	9471	9920	Hypothetical Protein
11	9926	10513	putative O-acetyl-ribose deacetylase
12	10522	10836	Hypothetical Protein
13	10842	11486	putative membrane protein
14	11747	12013	putative DNA primase
15	12262	12441	Hypothetical Protein
16	12450	13292	putative DNA adenine methylase
17	13308	13451	Hypothetical Protein
18	13460	14182	Hypothetical Protein
19	14175	14429	Hypothetical Protein

20	14510	15088	Hypothetical Protein
21	15088	15453	Hypothetical Protein
22	15440	15934	putative CMP deaminase
23	15944	16396	Hypothetical Protein
24	16399	17271	Hypothetical Protein
25	17264	18367	putative thymidylate synthase
26	18409	19119	Hypothetical Protein
27	19119	19766	Hypothetical Protein
28	20115	20354	Hypothetical Protein
29	20344	20748	Hypothetical Protein
30	20794	21498	Hypothetical Protein
31	21470	21940	Hypothetical Protein
32	21933	22892	Hypothetical Protein
33	22873	23736	Hypothetical Protein
34	23786	24121	Hypothetical Protein
35	24099	24386	Hypothetical Protein
36	24741	24896	Hypothetical Protein
37	24904	25317	Hypothetical Protein
38	25314	26033	Hypothetical Protein
39	26023	26334	Hypothetical Protein
40	26331	26690	Hypothetical Protein
41	26699	27451	Hypothetical Protein
42	27453	27713	Hypothetical Protein
43	27775	28416	Hypothetical Protein
44	28536	28901	Hypothetical Protein
45	28891	29250	Hypothetical Protein
46	29261	29728	Hypothetical Protein
47	29789	30382	Hypothetical Protein
48	30385	30597	Hypothetical Protein
49	30594	30863	Hypothetical Protein
50	30871	31302	Hypothetical Protein
51	31302	31829	Hypothetical Protein
52	31804	32643	Hypothetical Protein
53	32652	32906	Hypothetical Protein
54	32903	33304	putative ASCH domain-containing protein
55	33280	34038	Hypothetical Protein
56	34025	34555	Hypothetical Protein
57	34548	34865	Hypothetical Protein
58	34852	35175	Hypothetical Protein
59	35707	35952	Hypothetical Protein
60	35990	36574	putative bifunctional (p)ppGpp synthetase/guanosine-3
61	36578	36973	Hypothetical Protein
62	37181	37642	Hypothetical Protein

63	37839	38438	Hypothetical Protein
64	38435	38800	Hypothetical Protein
65	38797	39504	Hypothetical Protein
66	39506	40228	Hypothetical Protein
67	40265	41056	Hypothetical Protein
68	41095	41562	putative membrane protein
69	41559	41840	Hypothetical Protein
70	41848	42177	putative membrane protein
71	42188	42427	Hypothetical Protein
72	42445	43077	Hypothetical Protein
73	43077	43985	Hypothetical Protein
74	43996	44550	Hypothetical Protein
75	44547	45629	Hypothetical Protein
76	45626	46219	Hypothetical Protein
77	46219	47142	Hypothetical Protein
78	47142	47609	Hypothetical Protein
79	47623	48642	Hypothetical Protein
80	48669	50243	Hypothetical Protein
81	50244	50741	putative membrane protein
82	50824	51876	Hypothetical Protein
83	52039	53415	putative T1SS secreted agglutinin RTX
84	53434	54183	Hypothetical Protein
85	54180	54671	putative membrane protein
86	54673	54948	Hypothetical Protein
87	54984	55730	Hypothetical Protein
88	55733	56530	Hypothetical Protein
89	56523	56981	Hypothetical Protein
90	56983	57801	putative tail fibre protein
91	57813	58013	Hypothetical Protein
92	58058	61591	putative ILEI domain-containing protein
93	61600	63621	Hypothetical Protein
94	63634	64128	putative tail fibre protein
95	64142	64774	putative tail fibre protein
96	64785	65759	putative tail protein
97	65756	68077	Hypothetical Protein
98	68165	69235	Hypothetical Protein
99	69279	73802	Hypothetical Protein
100	73799	75262	putative baseplate wedge subunit protein
101	75259	75672	putative baseplate protein
102	75672	75962	putative baseplate spike
103	76655	76966	Hypothetical Protein
104	77703	79481	Hypothetical Protein
105	79491	79931	Hypothetical Protein

106	79916	80509	putative dTMP kinase
107	80514	80951	putative MmcB-like DNA repair protein/ transcription elongation factor
108	80992	81444	putative NUDIX hydrolase domain-containing protein
109	81441	81947	Hypothetical Protein
110	82245	82871	Hypothetical Protein
111	82884	83669	putative baseplate protein/tail-associated lysozyme
112	83666	85549	Hypothetical Protein
113	85553	85774	Hypothetical Protein
114	85781	86179	Hypothetical Protein
115	86179	86340	Hypothetical Protein
116	86445	87248	Hypothetical Protein
117	87248	90040	putative VgrG-like protein/endolysin
118	90040	90882	Hypothetical Protein
119	90875	91561	Hypothetical Protein
120	91572	92084	putative tail tube protein
121	92087	92755	Hypothetical Protein
122	92798	93313	putative tail tube protein
123	93328	95013	putative tail sheath protein
124	95071	95415	Hypothetical Protein
125	95417	96079	Hypothetical Protein
126	96151	96624	Hypothetical Protein
127	96716	97810	putative major capsid protein
128	97863	98567	putative structural protein
129	98637	100595	putative ATPase
130	100679	101782	Hypothetical Protein
131	101779	102600	putative prohead core protein protease/endolysin
132	102607	103029	Hypothetical Protein
133	103034	103921	Hypothetical Protein
134	103929	105029	putative glycosyl transferase
135	105040	105954	Hypothetical Protein
136	105954	107924	putative DNA ligase
137	107948	108871	Hypothetical Protein
138	108868	109710	Hypothetical Protein
139	109710	111641	Hypothetical Protein
140	111701	115663	Hypothetical Protein
141	115725	116879	Hypothetical Protein
142	116898	117872	Hypothetical Protein
143	117876	118640	Hypothetical Protein
144	118656	121739	putative major tail protein/T1SS secreted agglutinin RTX
145	121729	122373	Hypothetical Protein
146	122370	122996	Hypothetical Protein
147	123018	124694	putative tail sheath protein
148	124782	125828	Hypothetical Protein

149	125816	126670	Hypothetical Protein
150	126675	126884	Hypothetical Protein
151	126881	127513	Hypothetical Protein
152	127550	127831	Hypothetical Protein
153	127848	128231	Hypothetical Protein
154	128197	128535	Hypothetical Protein
155	128541	129434	putative DNA repair helicase
156	130511	131647	putative DNA repair helicase
157	131650	132213	Hypothetical Protein
158	132215	132643	Hypothetical Protein
159	132640	133815	Hypothetical Protein
160	133802	134071	Hypothetical Protein
161	134068	137223	putative DNA polymerase I
162	137318	137635	Hypothetical Protein
163	137638	138192	Hypothetical Protein
164	138236	144070	putative ATP-dependent DNA helicase
165	144081	144581	Hypothetical Protein
166	144594	145361	Hypothetical Protein
167	145361	145720	putative HNH family endonuclease
168	145727	146485	Hypothetical Protein
169	146546	147349	Hypothetical Protein
170	147402	148562	putative head to tail joining protein
171	148565	149662	Hypothetical Protein
172	149662	150939	Hypothetical Protein
173	151080	151742	Hypothetical Protein
174	151878	153008	putative recombination-related endonuclease
175	153123	153647	putative ssDNA binding protein
176	153694	155658	Hypothetical Protein
177	155655	157442	Hypothetical Protein
178	157453	157818	putative DUF2778 domain-containing protein
179	157818	158384	Hypothetical Protein
180	158403	159395	Hypothetical Protein
181	159398	159865	Hypothetical Protein
182	159862	160437	putative glycosyl hydrolase
183	160517	160840	Hypothetical Protein
184	160840	161553	Hypothetical Protein
185	161556	161807	Hypothetical Protein
186	161807	164107	putative exonuclease
187	164110	164310	Hypothetical Protein
188	164391	164627	Hypothetical Protein
189	164644	165078	Hypothetical Protein
190	165081	165758	putative SAM-dependent methyltransferase
191	165879	166358	Hypothetical Protein

192	166438	167658	putative DNA ploymerase III
193	168001	168576	Hypothetical Protein
194	168576	168908	Hypothetical Protein
195	168898	169875	Hypothetical Protein
196	169918	170385	Hypothetical Protein
197	170392	170886	Hypothetical Protein
198	170898	171362	Hypothetical Protein
199	171411	172499	Hypothetical Protein
200	172537	173754	Hypothetical Protein
201	174617	175168	Hypothetical Protein
202	175171	175347	Hypothetical Protein
203	175379	176416	Hypothetical Protein
204	176560	177108	putative holliday-junction resolvase
205	177121	177762	Hypothetical Protein
206	177821	178459	Hypothetical Protein
207	178456	180441	putative inverse autotransporter beta-barrel domain-containing protein
208	180563	181240	Hypothetical Protein
209	181276	182331	putative DNA primase
210	182397	182732	Hypothetical Protein
211	182770	183786	putatuve exonuclease
212	183794	184195	Hypothetical Protein
213	184649	185311	Hypothetical Protein
214	185314	185478	Hypothetical Protein
215	185490	185762	Hypothetical Protein
216	185759	186076	Hypothetical Protein
217	186119	186295	Hypothetical Protein
218	186298	186819	Hypothetical Protein
219	186816	187265	putative cyclic phosphodiesterase
220	187249	187530	Hypothetical Protein
221	187566	188603	Hypothetical Protein
222	188664	189869	putative ssDNA binding protein
223	189924	191429	putative RecA protein
224	191471	191872	Hypothetical Protein
225	191989	192768	Hypothetical Protein
226	192765	193343	Hypothetical Protein
227	193327	193860	Hypothetical Protein
228	193941	194189	Hypothetical Protein
229	194253	196070	Hypothetical Protein
230	196123	196716	Hypothetical Protein
231	196655	197296	Hypothetical Protein
232	197344	197985	Hypothetical Protein
233	197985	198302	Hypothetical Protein
234	198280	199449	putative methyltransferase

235	199505	199651	Hypothetical Protein
236	199630	200853	putative DNA adenine methylase
237	201030	201365	Hypothetical Protein
238	201428	201907	Hypothetical Protein
239	201907	202422	Hypothetical Protein
240	202412	202837	Hypothetical Protein
241	202806	203081	Hypothetical Protein
242	203081	203299	Hypothetical Protein
243	203301	204059	Hypothetical Protein
244	204326	204919	Hypothetical Protein
245	204903	205253	Hypothetical Protein
246	205262	205612	Hypothetical Protein
247	205624	205872	Hypothetical Protein
248	206043	206315	Hypothetical Protein
249	206380	206808	Hypothetical Protein
250	206868	207215	Hypothetical Protein
251	207212	207505	Hypothetical Protein
252	207515	208300	Hypothetical Protein
253	208297	208905	Hypothetical Protein
254	208902	209621	Hypothetical Protein
255	209623	210303	Hypothetical Protein
256	210352	211023	Hypothetical Protein
257	211020	211238	Hypothetical Protein
258	211660	212160	Hypothetical Protein
259	212162	212878	Hypothetical Protein
260	212878	213120	Hypothetical Protein
261	213240	213578	Hypothetical Protein
262	213581	214201	Hypothetical Protein
263	214198	214740	putative RNA 2
264	214843	215235	Hypothetical Protein
265	215245	215502	putative DksA/TraR family C4-type zinc finger protein
266	215505	215813	Hypothetical Protein
267	215816	215956	Hypothetical Protein
268	215956	216390	Hypothetical Protein
269	216409	216858	Hypothetical Protein
270	216894	217829	putative UvsE UV damage repair endonuclease
271	217820	217921	Hypothetical Protein
272	217930	218739	Hypothetical Protein
273	218750	219358	Hypothetical Protein
274	219359	221308	Hypothetical Protein
275	221361	222020	Hypothetical Protein
276	222156	222335	Hypothetical Protein
277	222339	222587	Hypothetical Protein

278	222591	223049	Hypothetical Protein
279	223052	223348	Hypothetical Protein
280	223361	223654	Hypothetical Protein
281	223709	224152	Hypothetical Protein
282	224155	224445	Hypothetical Protein
283	224445	225011	putative dUTPase
284	225389	225907	putative lytic transglycosylase
285	226054	226347	Hypothetical Protein
286	226363	226875	Hypothetical Protein
287	226888	227031	Hypothetical Protein
288	227074	227781	Hypothetical Protein
289	227781	228305	Hypothetical Protein
290	228302	228757	Hypothetical Protein
291	228758	228964	Hypothetical Protein
292	228971	229375	Hypothetical Protein
293	229426	230049	Hypothetical Protein
294	230150	230755	Hypothetical Protein
295	230752	230928	Hypothetical Protein
296	230925	231332	Hypothetical Protein
297	231343	232176	Hypothetical Protein
298	232193	232975	Hypothetical Protein
299	233130	233480	Hypothetical Protein
300	233631	234374	Hypothetical Protein
301	234430	234960	Hypothetical Protein
302	234967	235344	Hypothetical Protein
303	235344	235709	Hypothetical Protein
304	235995	237608	Hypothetical Protein
305	237681	238607	Hypothetical Protein
306	238607	238969	Hypothetical Protein
307	238978	239454	Hypothetical Protein
308	239426	240694	Hypothetical Protein
309	240694	243666	Hypothetical Protein
310	243721	244473	Hypothetical Protein
311	244496	245074	Hypothetical Protein
312	245279	246070	Hypothetical Protein
313	246248	246766	Hypothetical Protein
314	246860	247201	Hypothetical Protein
315	247320	249380	putative DNA topoisomerase IV/gyrase subunit B
316	249380	251062	putative DNA topoisomerase 4 subunit A
317	251216	252406	Hypothetical Protein
318	252396	252938	Hypothetical Protein
319	252910	253749	Hypothetical Protein
320	253811	254944	Hypothetical Protein

321	254952	255308	Hypothetical Protein

Table S5: Annotation table for JA33 (Genbank reference MH460462).

1.6 AD1 genome annotation table

ORF	Start	End	Annotation
1	34	1455	putative DNA helicase DnaB
2	1464	1706	hypothetical protein
3	1699	2496	hypothetical protein
4	2483	5311	putative terminase
5	5351	5566	hypothetical protein
6	5574	7517	putative portal protein
7	7517	7852	hypothetical protein
8	7849	8235	hypothetical protein
9	8264	8398	hypothetical protein
10	8398	8895	hypothetical protein
11	8892	9920	putative DNA polymerase I
12	9931	10473	hypothetical protein
13	10473	11066	putative O-acetyl-ADP-ribose deacetylase
14	11208	11423	hypothetical protein
15	11492	12181	putative membrane protein
16	12233	13003	putative methyltransferase
17	13015	14070	putative DUF1611 domain-containing protein
18	14063	14932	putative asparagine synthase
19	14934	15554	hypothetical protein
20	15567	15830	hypothetical protein
21	15838	16293	hypothetical protein
22	16290	16499	hypothetical protein
23	16529	16822	hypothetical protein
24	16830	17012	putative DNA primase
25	17265	17717	hypothetical protein
26	17732	18115	hypothetical protein
27	18354	19196	putative DNA adenine methylase
28	19204	19515	hypothetical protein
29	19568	20152	hypothetical protein
30	20152	20850	hypothetical protein
31	20843	21181	hypothetical protein
32	21159	21638	hypothetical protein
33	21619	22029	hypothetical protein
34	22014	22535	putative CMP deaminase
35	22561	23328	hypothetical protein
36	23339	23827	hypothetical protein

37	23814	23978	hypothetical protein
38	24020	24547	hypothetical protein
39	24551	25189	putative membrane protein
40	25164	25511	hypothetical protein
41	25511	25849	hypothetical protein
42	25849	26199	hypothetical protein
43	26199	27305	putative thymidylate synthase
44	27302	27952	hypothetical protein
45	27952	28842	hypothetical protein
46	28893	29228	hypothetical protein
47	29274	29984	putative transcriptional repressor
48	29986	32115	putative DNA-cytosine methyltransferase
49	32288	32827	hypothetical protein
50	32995	33405	hypothetical protein
51	33464	34369	hypothetical protein
52	34450	35109	hypothetical protein
53	35118	35471	hypothetical protein
54	35407	35931	hypothetical protein
55	35957	36265	hypothetical protein
56	36297	36764	hypothetical protein
57	36761	36961	hypothetical protein
58	36958	37356	putative ASCH domain-containing protein
59	37353	37568	hypothetical protein
60	37614	38039	hypothetical protein
61	38047	38529	hypothetical protein
62	38559	38885	hypothetical protein
63	38885	39400	hypothetical protein
64	39390	40064	putative GTP pyrophosphokinase
65	40039	40413	hypothetical protein
66	40385	40768	hypothetical protein
67	40740	41390	hypothetical protein
68	41541	41888	hypothetical protein
69	41842	42567	hypothetical protein
70	42634	43335	hypothetical protein
71	43335	44333	hypothetical protein
72	44391	44855	putative lipoprotein
73	44852	45163	hypothetical protein
74	45163	45471	putative membrane protein
75	45527	45766	hypothetical protein
76	45802	46401	hypothetical protein
77	46401	47303	hypothetical protein
78	47313	47846	hypothetical protein
79	47848	48897	hypothetical protein

80	48964	49488	hypothetical protein
81	49485	50561	hypothetical protein
82	50571	51149	hypothetical protein
83	51149	52051	hypothetical protein
84	52051	52506	hypothetical protein
85	52508	53596	hypothetical protein
86	53598	54554	hypothetical protein
87	54554	55051	putative membrane protein
88	55119	55613	hypothetical protein
89	56052	57002	hypothetical protein
90	57012	58055	hypothetical protein
91	58065	58700	putative structural protein
92	58761	59342	hypothetical protein
93	59354	59575	hypothetical protein
94	59587	60183	hypothetical protein
95	60241	61860	hypothetical protein
96	61885	62628	hypothetical protein
97	62625	63134	putative membrane protein
98	63140	63424	hypothetical protein
99	63489	64352	hypothetical protein
100	64388	65212	hypothetical protein
101	65196	65672	hypothetical protein
102	65674	66495	putative tail fibre protein
103	66507	66710	hypothetical protein
104	66720	69593	putative ILEI domain-containing protein
105	69637	71661	hypothetical protein
106	71671	72162	putative tail fibre protein
107	72172	72804	putative tail fibre protein
108	72804	74243	putative tail protein
109	74240	76579	hypothetical protein
110	76665	81200	hypothetical protein
111	81197	82660	putative baseplate wedge subunit protein
112	82662	82787	putative baseplate wedge subunit
113	82789	83211	putative baseplate protein
114	83211	83501	putative baseplate spike protein
115	84958	86721	hypothetical protein
116	86730	87140	hypothetical protein
117	87161	87775	putative dTMP kinase
118	87785	88276	putative MmcB-like DNA repair protein
119	88266	88724	putative NUDIX hydrolase
120	88721	89224	hypothetical protein
121	89263	89553	hypothetical protein
122	89550	90275	hypothetical protein

123	90286	91074	putative baseplate protein
124	91071	92966	hypothetical protein
125	92969	93403	hypothetical protein
126	93403	93696	hypothetical protein
127	93681	94487	hypothetical protein
128	94500	97139	putative VGRG protein
129	97139	97918	hypothetical protein
130	97984	98667	hypothetical protein
131	98678	99190	putative tail tube protein
132	99193	99867	hypothetical protein
133	99919	100431	putative tail tube protein
134	100443	102128	putative tail sheath protein
135	102185	102529	hypothetical protein
136	102529	103218	hypothetical protein
137	103284	103706	hypothetical protein
138	103759	104859	putative major capsid protein
139	104919	105626	putative structural protein
140	105682	107676	hypothetical protein
141	107753	108880	hypothetical protein
142	108880	109659	putative prohead core protein protease
143	109670	110080	hypothetical protein
144	110082	110849	hypothetical protein
145	110818	112026	putative glycosyl transferase
146	112088	113005	hypothetical protein
147	113008	114954	putative DNA ligase
148	114994	116853	hypothetical protein
149	116916	121043	hypothetical protein
150	121099	122280	hypothetical protein
151	122299	123285	hypothetical protein
152	123293	124078	hypothetical protein
153	124089	128099	putative major tail protein
154	128096	128728	hypothetical protein
155	128739	129377	hypothetical protein
156	129412	130107	hypothetical protein
157	130155	131831	putative tail sheath protein
158	131946	133031	hypothetical protein
159	133034	133900	hypothetical protein
160	133897	134091	hypothetical protein
161	134101	134721	hypothetical protein
162	134742	135104	hypothetical protein
163	135120	135491	hypothetical protein
164	135481	135804	hypothetical protein
165	135842	136333	hypothetical protein

166	136336	136818	hypothetical protein
167	136818	138419	putative DNA repair helicase
168	138422	138967	hypothetical protein
169	138967	139401	hypothetical protein
170	139410	140588	hypothetical protein
171	140554	140847	hypothetical protein
172	140844	144005	putative DNA polymerase I
173	144095	145072	hypothetical protein
174	145082	145633	hypothetical protein
175	145677	151523	putative ATP-dependent DNA helicase
176	151523	152017	hypothetical protein
177	152028	152810	hypothetical protein
178	152807	153172	putative HNH family endonuclease
179	153206	154024	hypothetical protein
180	154084	154884	hypothetical protein
181	154931	156094	putative head to tail joining protein
182	156096	157127	hypothetical protein
183	157202	158485	hypothetical protein
184	158658	159347	hypothetical protein
185	159466	160539	putative recombination related endonuclease
186	160580	161335	hypothetical protein
187	161339	161845	putative ssDNA binding protein
188	161885	162250	putative DUF2778 domain-containing protein
189	162332	162841	hypothetical protein
190	162842	163753	hypothetical protein
191	163772	164233	hypothetical protein
192	164371	164706	hypothetical protein
193	164709	165281	putative glycosyl hydrolase
194	165318	166163	hypothetical protein
195	166250	166591	putative membrane protein
196	166567	166884	hypothetical protein
197	166886	167599	hypothetical protein
198	167602	167922	hypothetical protein
199	167922	170252	putative exonuclease
200	170252	170452	hypothetical protein
201	170452	170664	hypothetical protein
202	170744	171265	hypothetical protein
203	171265	171714	hypothetical protein
204	171845	172381	hypothetical protein
205	172487	173776	putative DNA polymerase III
206	173919	174116	hypothetical protein
207	174591	175178	hypothetical protein
208	175178	175522	hypothetical protein

209	175527	176099	hypothetical protein
210	176099	177073	hypothetical protein
211	177085	177519	hypothetical protein
212	177611	177805	hypothetical protein
213	177845	178369	hypothetical protein
214	178373	179425	hypothetical protein
215	180393	181052	hypothetical protein
216	181062	182183	hypothetical protein
217	182283	182831	putative holliday junction resolvase
218	182839	183204	hypothetical protein
219	183251	183943	hypothetical protein
220	183936	185705	putative inverse autotransporter beta-barrel domain-containing protein
221	185759	186409	hypothetical protein
222	186466	186645	hypothetical protein
223	186647	187705	putative DNA primase
224	187770	188105	hypothetical protein
225	188068	189153	putative exonuclease
226	189231	189560	hypothetical protein
227	189568	190032	hypothetical protein
228	189992	190525	hypothetical protein
229	190458	191114	hypothetical protein
230	191120	191401	hypothetical protein
231	191382	191813	hypothetical protein
232	191813	192028	hypothetical protein
233	192031	192759	hypothetical protein
234	192752	193285	hypothetical protein
235	193278	193745	putative cylic phosphodiesterase
236	193797	194108	hypothetical protein
237	194154	195191	hypothetical protein
238	195242	196516	putative ssDNA binding protein
239	196574	199099	putative RecA protein
240	199149	199568	hypothetical protein
241	199668	200369	hypothetical protein
242	200546	201472	hypothetical protein
243	201517	202284	hypothetical protein
244	202718	204256	hypothetical protein
245	204427	205131	hypothetical protein
246	205236	205475	hypothetical protein
247	205532	206035	hypothetical protein
248	206098	206712	hypothetical protein
249	206761	207021	hypothetical protein
250	207100	207468	hypothetical protein
251	207465	207710	hypothetical protein

252	207703	208065	hypothetical protein
253	208154	208639	hypothetical protein
254	208627	208944	hypothetical protein
255	209037	209354	hypothetical protein
256	209419	209772	hypothetical protein
257	209838	210242	hypothetical protein
258	210300	210752	putative XRE family transcriptional regulator
259	210745	211284	hypothetical protein
260	211297	211737	hypothetical protein
261	211734	212177	hypothetical protein
262	212259	212486	hypothetical protein
263	212483	213538	hypothetical protein
264	213535	213744	hypothetical protein
265	213737	214270	putative RNA NAD 2
266	214273	214686	hypothetical protein
267	214742	216229	putative radical SAM superfamily protein
268	216244	216861	hypothetical protein
269	216863	217219	hypothetical protein
270	217219	217698	hypothetical protein
271	217689	217952	putative DksA/TraR family C4-type zinc finger protein
272	217955	218383	hypothetical protein
273	218380	218565	hypothetical protein
274	218565	218882	hypothetical protein
275	218879	219076	hypothetical protein
276	219073	219426	hypothetical protein
277	219426	220145	hypothetical protein
278	220155	220784	hypothetical protein
279	220781	221152	hypothetical protein
280	221154	221912	hypothetical protein
281	221887	222090	hypothetical protein
282	222077	222628	hypothetical protein
283	222687	223667	putative UV damage repair endonuclease
284	223756	224058	hypothetical protein
285	224060	224323	hypothetical protein
286	224752	225003	hypothetical protein
287	225078	225590	hypothetical protein
288	225590	225877	hypothetical protein
289	225893	227065	hypothetical protein
290	227178	227480	hypothetical protein
291	227489	228043	hypothetical protein
292	228056	228514	hypothetical protein
293	228525	229094	putative dUTPase
294	229087	229302	hypothetical protein

295	230406	231092	hypothetical protein
296	231161	232102	hypothetical protein
297	232180	232884	hypothetical protein
298	232890	233117	hypothetical protein
299	233120	233428	hypothetical protein
300	233428	234003	hypothetical protein
301	234056	235657	hypothetical protein
302	235693	236127	hypothetical protein
303	236136	236435	hypothetical protein
304	236542	237006	hypothetical protein
305	237008	237703	hypothetical protein
306	237703	237894	hypothetical protein
307	238035	238517	hypothetical protein
308	238522	238737	hypothetical protein
309	238992	239723	hypothetical protein
310	239723	240172	hypothetical protein
311	240190	240342	hypothetical protein
312	240505	242136	hypothetical protein
313	242225	243457	hypothetical protein
314	243454	243834	hypothetical protein
315	243831	244292	hypothetical protein
316	244282	245562	hypothetical protein
317	245590	248613	hypothetical protein
318	248671	249501	hypothetical protein
319	249602	250147	hypothetical protein
320	250297	251118	hypothetical protein
321	251337	251873	hypothetical protein
322	251971	252471	hypothetical protein
323	252496	253236	hypothetical protein
324	253374	255404	putative DNA gyrase subunit B
325	255406	257052	putative DNA topoisomerase 4 subunit A
326	257399	258673	hypothetical protein
327	258666	259208	hypothetical protein
328	259141	260013	putative DNA topoisomerase 4 subunit A
329	260068	261207	hypothetical protein
330	261225	261596	hypothetical protein

Table S6: Annotation table for AD1 (Genbank reference MH460463).
